# False discovery rate control in two-stage designs

**DOI:** 10.1186/1471-2105-13-81

**Published:** 2012-05-06

**Authors:** Sonja Zehetmayer, Martin Posch

**Affiliations:** 1 Center for Medical Statistics, Informatics, and Complex Systems, Medical University of Vienna, Vienna, Austria; 2 European Medicines Agency (EMA), London, UK

## Abstract

**Background:**

For gene expression or gene association studies with a large number of hypotheses the number of measurements per marker in a conventional single-stage design is often low due to limited resources. Two-stage designs have been proposed where in a first stage promising hypotheses are identified and further investigated in the second stage with larger sample sizes. For two types of two-stage designs proposed in the literature we derive multiple testing procedures controlling the False Discovery Rate (FDR) demonstrating FDR control by simulations: designs where a fixed number of top-ranked hypotheses are selected and designs where the selection in the interim analysis is based on an FDR threshold. In contrast to earlier approaches which use only the second-stage data in the hypothesis tests (pilot approach), the proposed testing procedures are based on the pooled data from both stages (integrated approach).

**Results:**

For both selection rules the multiple testing procedures control the FDR in the considered simulation scenarios. This holds for the case of independent observations across hypotheses as well as for certain correlation structures. Additionally, we show that in scenarios with small effect sizes the testing procedures based on the pooled data from both stages can give a considerable improvement in power compared to tests based on the second-stage data only.

**Conclusion:**

The proposed hypothesis tests provide a tool for FDR control for the considered two-stage designs. Comparing the integrated approaches for both selection rules with the corresponding pilot approaches showed an advantage of the integrated approach in many simulation scenarios.

## Background

Modern experimental techniques in genetic research such as microarray experiments or gene association studies produce high dimensional data and often thousands of hypotheses are tested simultaneously to identify genetic markers. Due to limited resources, the number of measurements per marker in a conventional single-stage design is often low. Two-stage designs have been proposed where in a first stage promising markers are identified from the set of all markers considered initially. Thus, hypotheses corresponding to unpromising markers can be dropped in the interim analysis such that the second stage is performed with the reduced set of selected hypotheses. Given limited total resources or budgets, this allows the allocation of a larger number of observations to more promising hypotheses. It has been shown that such sequential procedures are typically considerably more powerful than single-stage designs [1-8].

An important problem when drawing inference from data produced by such designs is the construction of hypothesis tests that control the False Discovery Rate (FDR). While the construction of such test procedures is straightforward if only the second-stage data is used for testing, tests that make use of the data from both stages need to account for the specific selection rule used to select hypotheses for the second stage.

For two-stage procedures where in an interim analysis all hypotheses with an unadjusted first-stage *p*-value below a pre-fixed selection boundary *γ*_1_are selected for the second stage, hypothesis tests based on the pooled data from both stages have been proposed that control FDR or the familywise error rate [7] (see [8] for a generalization to multistage designs). Selecting all hypotheses whose first-stage *p*-value lies below a fixed threshold has several implications. First, the number of hypotheses selected for the second stage is a random variable unknown a priori, which is an obstacle for researchers if resources are limited and the number of markers for which further measurements can be collected cannot be arbitrarily increased. Second, because rather large thresholds need to be applied in the interim analysis in order not to miss alternative hypotheses in spite of the small sample size, the fixed threshold rule will, with a high probability, select some hypotheses even under the global null hypothesis, even though resources could be saved in this scenario by stopping the experiment for futility at the interim analysis.

In this work we propose statistical tests to control the FDR in two-stage designs with selection rules that are not based on a fixed threshold for the first-stage *p*-values. First, we consider designs where a fixed number of hypotheses is selected for the second stage (FNS design) [4]. The selected hypotheses are the top-ranked hypotheses according to the first stage *p*-values. Second, we consider two-stage designs where selection is based on a fixed FDR threshold *α*_1_ in the interim analysis (FDRS design). All hypotheses that can be rejected with a test controlling the FDR at level *α*_1_ are selected for the second stage [9]. For the FNS design the number of continued hypotheses is deterministic and the procedure continues to the second stage, regardless of the actual effect sizes observed. For the FDRS design in contrast, the number of selected hypotheses is a random variable. Furthermore, under the global null hypothesis (where the FDR coincides with the familywise error rate) with probability 1−*α*_1_ no hypothesis can be rejected with the interim test at FDR level *α*_1_and the trial is stopped for futility.

A simple approach to construct hypothesis tests controlling the FDR for two-stage designs is to consider tests based on the second-stage data only. Standard multiple testing procedures applied to the second-stage data will control the FDR. For the FDRS design Benjamini and Yekutieli [9] showed that the nominal level applied at the second stage may be even increased taking into account the interim selection threshold. However, these approaches do not make full use of the available data because the first-stage observations are used for selection only. We construct tests for the FNS and FDRS designs that are based on sufficient test statistics of the data from both stages (the “integrated approach”). These tests are designed in analogy to group sequential tests and appear to control the FDR well if the number of hypotheses tested is large enough.

The paper is structured as follows: in the next section the testing problem and the selection rules are introduced. Then the results of a simulation study which investigates the actual FDR and compares the mean number of rejected alternatives of the integrated approach to the pilot approach are presented. Finally, a real data example and a short discussion are given.

## Methods

### The test problem

We consider an experiment to test *m* two-sided null hypotheses *H*_0*i*_: *μ*_*i*_=0 versus *H*_1*i*_: *μ*_*i*_≠0, i=1,…,m, for the mean of independent, normally distributed observations with variances $\sigma_{i}^{2}$, i=1,…,m. More general distributional scenarios are discussed at the end of this section.

To adjust for multiple testing we aim to control the FDR of the experiment. The FDR [10] is defined as the expectation of the proportion of erroneously rejected hypotheses *V * among all rejected hypotheses *R*, FDR=E(V/max{R,1}). We apply the step-up Benjamini-Hochberg (BH) procedure to control the FDR at level *α*. Denote the ordered *p*-values of the *m* hypotheses by $p_{\left(1\right)}\leq p_{\left(2\right)}\leq \cdots \leq p_{\left(m\right)}$ and let *d* = argmax_*i*_ { *p*_ (*i*)_ ≤ *iα* / *m* } denote the index of the largest *p*-value *p*_(*i*)_ smaller than or equal to *iα*/*m*. Then the BH-procedure rejects all hypotheses *H*_0*i*_such that *p*_(*i*)_≤*dα*/*m*. For single-stage tests it has been shown by Benjamini and Yekutieli [11] that the BH-procedure controls the FDR at level *Π*_0_*α*, if the subset of test statistics corresponding to true null hypotheses are independent or positively regression dependent. Here, *Π*_0_ denotes the (unknown) proportion of true null hypotheses among the *m* tested null hypotheses. Furthermore, the FDR is asymptotically controlled (for increasing number of hypotheses) if the limiting fraction of true null hypotheses is less than one and the test statistics are weakly dependent such that their empirical distribution functions converge almost surely (and some additional technical conditions hold) [12]. In the following we assume distributional scenarios where the BH-procedures controls the FDR.

### The two-stage procedure

In the first-stage for each hypothesis *n*_1_observations are collected. Then an interim analysis is performed and for each hypothesis a two-sided first-stage *p*-value $p_{i}^{\left(1\right)}=2(1-\text{Φ}(|z_{i}^{\left(1\right)}|)$, i=1,…,m, is calculated, where $z_{i}^{\left(1\right)}$ denotes the standardized first-stage mean for hypothesis *i* and Φ the cumulative distribution function of the standard normal distribution. The first-stage *p*-values are ranked according to their magnitude and the *m*_2_ hypotheses with the smallest *p*-value are selected for the second stage. The number of selected hypotheses *m*_2_ can be either a pre-fixed number or may depend on the first-stage results. Below we consider several choices for *m*_2_. In a second stage for each selected hypothesis *n*_2_ observations are collected. *n*_2_ is assumed to be fixed and does not depend on the number of selected hypotheses. We consider two different approaches to arrive at the final test decision: the “integrated approach”, where the test decision is based on the combined data of both stages and the “pilot approach”, where the test decision is based on the second-stage data only.

In the following we introduce several rules to determine the number of selected hypotheses *m*_2_.

### Selection rules for two-stage designs

#### Selection according to a prefixed selection boundary *γ*_1_

Two-stage designs have been proposed [2,7,8] where a selection boundary *γ*_1_ is pre-specified and in the interim analysis all hypotheses with a first-stage *p*-value smaller than *γ*_1_ are selected for the second stage. Then

(None) $$m_{2}=\overset{m}{\underset{i=1}{\sum }}1_{\left\{p_{i}^{\left(1\right)}\leq \gamma_{1}\right\}},$$

where **1** is the indicator function which equals 1 if the condition in the parentheses is satisfied and 0 otherwise. Thus, *m*_2_ is a random variable.

#### Pre-fixed number of hypotheses selected for the second stage - FNS design

With this rule the value of *m*_2_ is fixed a priori and the *m*_2_ hypotheses with the smallest first-stage *p*-values are selected for the second stage. We denote this procedure FNS design (Fixed Number Selection).

#### Selection based on an FDR threshold - FDRS design

In this approach all hypotheses which are significant according to the BH-procedure at a prefixed level *α*_1_>*α*in the interim analysis are selected for the second stage. Thus, the number of selected hypotheses *m*_2_ is a random variable which depends on the first-stage results. If no hypothesis can be rejected at level *α*_1_ in the interim analysis and thus be carried over to the second stage, *m*_2_ is set to zero. In this case the whole experiment is stopped. Note that under the global null hypothesis, i.e. in the setting where all null hypotheses are true and thus *Π*_0_=1, this occurs with a probability of 1−*α*_1_.

### FDR control

In the subsections below we review the FDR controlling test procedure for two-stage designs where hypotheses are selected based on a prefixed selection boundary applied to the first-stage *p*-values. Both pilot and integrated designs are considered. We then propose generalizations of these test procedures to FNS and FDRS designs, showing that for each of the designs a corresponding data dependent selection boundary for the first-stage *p*-values can be defined that converges under suitable assumptions almost surely to a fixed value. The results are derived for independent data. However, in genetic data dependence of test statistics is frequently observed and even weak dependence may be a strong assumption. In the simulation study we thus investigate the performances of the procedures for several correlation structures.

#### Selection according to a prefixed selection boundary *γ*_1_

##### Pilot approach

In the pilot approach the final test statistics are based on data from the second stage only. The first-stage data are used for selecting promising hypotheses only. To control the FDR of the experiment, at the end of the trial for each hypothesis a two-sided *p*-value $p_{i}^{\left(2\right)}=2(1-\text{Φ}(|z_{i}^{\left(2\right)})|)$, $i=1,\ldots ,m_{2}$, is calculated, where $z_{i}^{\left(2\right)}$ denotes the standardized mean of the second-stage observations for hypothesis *i*. Then the BH-procedure is applied to these *p*-values which are based on the second-stage data only. Because the first-stage observations are used only for selection and do not enter the final test statistics, the BH-procedure controlling the FDR at the second stage controls the FDR overall.

##### Integrated approach

If the data from both stages are to be used in the final test decision, one can account for the selection in the interim analysis by calculating sequential *p*-values *p*_*i*_, i=1…,m, based on the monotonic ordering of the sample space [13]. If $p_{i}^{\left(1\right)}>\gamma_{1}$ then the two-sided sequential *p*-value is defined as

(1) $$p_{i}=p_{i}^{\left(1\right)}=P_{H_{o}}\left(\left|Z_{i}^{\left(1\right)}|\geq |z_{i}^{\left(1\right)}\right|\right)$$

and if $p_{i}^{\left(1\right)}\leq \gamma_{1}$ it is given by

(2) $$p_{i}=P_{H_{o}}\left(\left\{|Z_{i}|\geq |z_{i}|\}\cap \{|Z_{i}^{\left(1\right)}|\geq c_{1-\gamma_{1}/2}\right\}\right),$$

where *Z*_*i*_ denotes the standardized overall mean of the observations from both stages and $z_{i}^{\left(1\right)}$ the standardized mean of the observations in the first stage. Furthermore, $z_{i},z_{i}^{\left(1\right)}$ denote realizations of the random variables $z_{i},z_{i}^{\left(1\right)}$ and $c_{1-\gamma_{1}/2}$ the (1−*γ*_1_/2)-quantile of the standard normal distribution. If the stopping criterion is satisfied the sequential *p*-value is just the classical fixed sample *p*-value calculated from the first-stage observations. Otherwise, the calculation of the sequential *p*-value involves the numerical solution of an integral (see the Appendix). Finally, the BH-procedure is applied to the sequential *p*-values $p_{1},\ldots ,p_{m}$.

In a two-stage procedure with fixed per hypothesis sample sizes *n*_1_, *n*_2_ and a fixed selection boundary *γ*_1_ the sequential *p*-values are uniformly distributed under the null hypothesis [13].

If for the subset of true null hypotheses the observations are independent across hypotheses such that the sequential *p*-values are independent as well, it follows that the FDR is controlled in such a two-stage design. As described above FDR control holds also if the sequential *p*-values corresponding to true null hypotheses are positive regression dependent [11].

Next we extend the test procedure to the FNS and FDRS design.

#### FNS design

##### Pilot approach

For the FNS selection rule the FDR control with the pilot approach is straightforward: the BH-procedure can be applied to the second-stage *p*-values of the *m*_2_ selected hypotheses. Because the first-stage data do not enter the final test statistics, FDR control is guaranteed under the assumption of positive regression dependency.

##### Integrated approach

To utilize the data from both stages for the final test decision, we propose to compute sequential *p*-values in analogy to (2), replacing the fixed selection boundary *γ*_1_ (which is not defined for the FNS design) by the value of the largest first-stage *p*-value of all selected hypotheses, i.e., setting $\gamma_{1}=p_{\left(m_{2}\right)}$. Thus, the threshold *γ*_1_ is now data dependent. Because this is not accounted for in the calculation of the sequential *p*-values, they may no longer be uniformly distributed under the null hypothesis and it is not obvious if the FDR is still controlled. However, if the observations are sufficiently independent across hypotheses, such that the empirical distribution functions of the first-stage test statistics converge almost surely as the number of tested hypotheses increases (and some additional technical conditions hold, see the Appendix), *γ*_1_ converges almost surely to a fixed number. Thus, asymptotically *γ*_1_ is deterministic and for large *m* and *m*_2_ the procedure becomes similar to the method with a prefixed selection boundary.

Note that with the integrated two-stage testing procedure, hypotheses that have not been selected in the interim analysis can in principle be rejected in the final test. Especially, if *m*_2_ is small compared to the number of hypotheses for which the alternative holds and the effect sizes are large, hypotheses that were not selected at the interim analysis can be rejected at the end because for every hypothesis a sequential *p*-value is calculated (even for non-selected ones). Rejection of non-selected hypotheses can occur in an overpowered FNS design where only few hypotheses are selected, but even the sequential *p*-values corresponding to non-selected alternative hypotheses are small enough to lead to a rejection. If such rejections occur, this is an indication that the first-stage sample size has been chosen too large and no second-stage sample would have been needed to reach sufficient power. While the efficiency of such a design can be improved by choosing appropriate first-stage sample sizes and selection rules, the control of the FDR is not affected.

#### FDRS design

##### Pilot approach

As for the FNS selection rule, if the BH-procedure is applied at nominal level *α* to the second-stage *p*-values of the *m*_2_ selected hypotheses (computed from the observations of the second stage only), FDR control is guaranteed. However, as Benjamini and Yekutieli [9] showed, if, in a first stage, hypotheses are selected that can be rejected with the BH-procedure at nominal level *α*_1_, and, in a second stage, the selected hypotheses are tested at nominal level *α*_2_, the FDR of the second-stage test is actually controlled at level *α*_1_*α*_2_*Π*_0_, given the test statistics at each stage are positively regression dependent [9]. Thus, if in the second stage the nominal level *α*/*α*_1_ is applied, the FDR is still controlled at level *Π*_0_*α*. In the following we consider the latter, improved procedure.

#### Integrated approach

Similar to the FNS rule we propose to compute sequential *p*-values in analogy to (2) to utilize the data from both stages for the final test decision.

Again, the resulting threshold *γ*_1_ is data dependent: we set *γ*_1_=*m*_2_*α*_1_/*m*where *m*_2_ is a random variable. Then *γ*_1_ is approximately equal to the largest first-stage *p*-value of all selected hypotheses. Thus the sequential *p*-values may no longer be uniformly distributed under the null hypothesis and FDR control is in question. However, the following argument gives a heuristic for FDR control when the number of hypotheses is large. If for a positive proportion of hypotheses the alternative holds the empirical distribution functions of the first-stage test statistics converge almost surely as the number of tested hypotheses increases (and some additional technical conditions hold, see the Appendix), it can be shown that *γ*_1_ converges almost surely. Hence, in these settings *γ*_1_ is asymptotically deterministic.

Under the global null hypothesis *γ*_1_ does not converge and simulations (see the Results section) show that the FDR is actually inflated. Therefore, we suggest the following modification of the test procedure. Let *m*_*s*_ > 0 denote a positive constant. In cases where less than *m*_*s*_ hypotheses are selected by the FDRS selection rule the threshold *γ*_1_ used in (2) is set to the *m*_*s*_-smallest first-stage *p*-value, thus $\gamma_{1}=\max(\overset{\left(1\right)}{\underset{\left(m_{2}\right)}{p}},p_{\left(m_{s}\right)}^{\left(1\right)})$. Note that this modification increases the first-stage critical boundary used in the calculation of the sequential *p*-value.

### Generalizations to other testing problems

The procedure can be directly generalized to two group comparisons, replacing the standardized means by the standardized mean between group differences. More generally, the sequential *p*-value can be computed as in (2) if (under the null hypothesis) the cumulative test statistics follow a multivariate normal distribution with mean zero and variance one. In the actual computation based on (3) the term *n*_1_/(*n*_1_ + *n*_2_) (resp. *n*_2_/(*n*_1_ + *n*_2_)) is then replaced by the correlation *ρ*(resp. 1−*ρ*). For example, for testing problems as the comparison of rates, or the analysis of co-variances, the standardized means can be replaced by standardized efficient score statistics that are asymptotically normally distributed. The correlation between these test statistics is determined by the information fractions (see [14]).

## Results

First we investigate the actual FDR of the proposed testing procedures for the FNS and FDRS selection rules. Additionally, to quantify the advantage in power of the integrated approach compared to the pilot approach, we report the mean number of rejected alternatives under different scenarios. We consider the one-sample *z*-test for *m* two-sided null hypotheses *H*_0*i*_: *μ*_*i*_=0 versus *H*_1*i*_: *μ*_*i*_≠0, i=1,…,m, for the mean of normally distributed observations with nominal significance level *α*=0.05. The simulations are performed for a wide range of scenarios. For a detailed description see Additional file 1.

In the following we assume independence of test statistics across hypotheses. However, because this assumption is often not satisfied in genetic data, we also report simulations assuming several correlation structures.

All computations were performed using the statistical language R [15]. R-code to reanalyse the real data application is available for download from the authors’ web page http://statistics.msi.meduniwien.ac.at/index.php?page=pageszfnr.

### Simulation results for the FNS procedure

#### Control of the error rate

Integrated approach: In all simulated scenarios the FDR is well controlled if *m*_2_ > 5 (see Additional file 1). Only if a smaller *m*_2_ is chosen, the FDR may be inflated up to 0.11.

**Figure 1:**
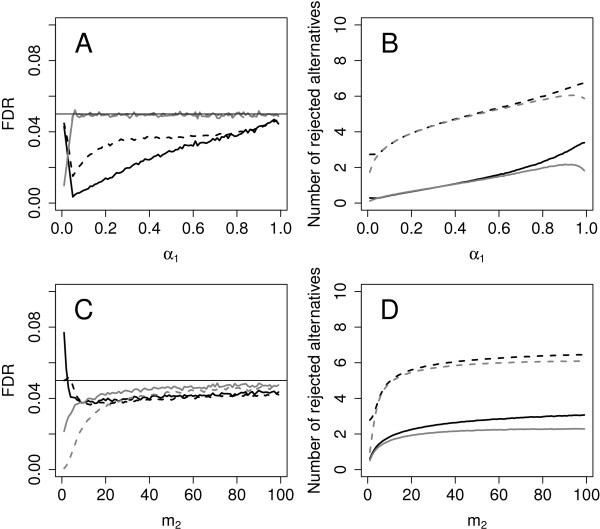
**Power values and error rates.(A)** and **(C)** show the actual FDR for the FDRS and the FNS design, respectively, **(B)** and **(D)** the corresponding mean number of rejected alternatives for *n*_1_=6, *n*_2_=12, *α*=0.05, *m*=1000, *Π*_0_=0.99, *m*_*s*_=6. The effect sizes are *Δ*=1 (solid line) or *Δ*=1.6 (dotted line). The integrated approach is depicted in black, the pilot approach in grey (50000 simulation runs per scenario)

A heuristic explanation for this inflation is that for very small *m*_2_ the *p*-value threshold corresponding to the FNS design, $p_{\left(m_{2}\right)}$, is close to zero. For such low *p*-value thresholds even small changes may lead to large changes in the sequential *p*-value (2) and the approximation based on a fixed threshold is poor. Figure 1C illustrates the decrease of the FDR with increasing *m*_2_ for two particular scenarios.

Pilot approach: For the pilot approach the FDR is controlled in all scenarios.

#### Mean number of rejected hypotheses

Table 1 shows the mean number of rejected alternatives for selected scenarios for the integrated approach controlling the FDR and the improvement in percent compared to the pilot approach. While the simulation study investigating FDR control covers *Π*_0_ values from 0.5 to 1 for the investigation of the power we consider settings where alternative hypotheses are sparse. These are settings where the advantage of two-stage designs that select promising hypotheses at interim analysis is expected to be largest.

**Table 1:** FNS design

		***m=*1000**		***m=*10000**		***m=*100000**	
_***m*2**_	_***Π*0**_	***Δ=*1**	***Δ=*1.6**	***Δ=*1**	***Δ=*1.6**	***Δ=*1**	***Δ=*1.6**
0.01*m*	.95	6.1 (17%)	15.4 (58%)	58.7 (19%)	141.9 (46%)	583.7 (19%)	1406.8 (45%)
0.01*m*	.99	1.8 (13%)	5.0 (2%)	13.3 (19%)	41.9 (3%)	126.8 (20%)	407.4 (3%)
0.05*m*	.95	12.5 (21%)	26.7 (5%)	117.6 (23%)	260.3 (5%)	1166.9 (23%)	2590.3 (5%)
0.05*m*	.99	2.8 (25%)	6.2 (4%)	19.8 (35%)	53.8 (6%)	188.4 (36%)	523.7 (6%)
0.1*m*	.95	14.9 (27%)	30.1 (6%)	139.9 (29%)	293.9 (7%)	1388.2 (29%)	2926.5 (7%)
0.1*m*	.99	3.1 (33%)	6.5 (6%)	22.0 (47%)	57.0 (9%)	209.9 (49%)	554.7 (9%)

In all scenarios the integrated approach rejects more or the same number of alternative hypotheses than the pilot approach. The increase in rejections is up to 59%. Figure 1D shows the impact of *m*_2_ on the mean number of rejected alternatives for the integrated (black lines) and the pilot approach (grey lines): For *Δ*=1 (solid lines) and very small *m*_2_, the number of rejected alternatives is very small but it clearly increases with *m*_2_. Here the difference to the pilot design is more distinct. For *Δ*=1.6 the advantage of the integrated approach is only moderate.

### Simulation results for the FDRS procedure

#### Control of the error rate

Integrated approach: For the original procedure (without the modified critical value) and *Π*_0_ < 0.8 the FDR is controlled for all considered values of *m*, *α*_1_ and *Δ* (data not shown). For larger values of *Π*_0_ the FDR may be inflated, especially if the effect size under the alternative is low such that the expected number of selected hypotheses for the second stage is very small. The inflation is, however, moderate and the maximal FDR over all simulation scenarios is 0.073 instead of the nominal 0.05.

The simulations for the modified procedure show that across all scenarios the FDR is controlled for *m*_*s*_=6 (see Figure 1A for an example and Additional file 1 for all scenarios). Therefore, in the following we only report results of the modified procedure with *m*_*s*_=6. Note that for some of the parameter values the modified procedure is strictly conservative.

For the pilot approach FDR control follows by theoretical arguments in [9] and is confirmed by the simulation study (within the simulation error, see Figure 1A).

#### Mean number of rejected hypotheses

Table 2 and Figure 1B show that the number of rejected alternatives increases with *α*_1_ as expected. For small values of *α*_1_, the pilot and the integrated approach have similar power values. In some settings for lower *m* the pilot design even slightly outperforms the integrated design. This is due to the fact that the modified FDRS procedure may be strictly conservative, especially if the number of selected hypotheses is low.

**Table 2:** FDRS design

		***m=*1000**		***m=*10000**		***m=*100000**	
***α***_**1**_	***Π***_**0**_	***Δ=*1**	***Δ=*1.6**	***Δ=*1**	***Δ=*1.6**	***Δ=*1**	***Δ=*1.6**
0.1	.95	2.5 (-1 %)	18.3 (0%)	17.7 (1%)	171.7 (0%)	166.2 (0%)	1703.0 (0%)
	.99	0.4 (-4%)	3.3 (0%)	1.3 (-3%)	23.1 (0%)	7.4 (0%)	219.7 (0%)
0.2	.95	4.3 (1%)	21.7 (1%)	34.1 (2%)	206.4 (0%)	328.6 (2%)	2047.1 (0%)
	.99	0.6 (-3%)	3.9 (0%)	2.3 (0%)	28.9 (0%)	15.8 (1%)	275.7 (0%)
0.5	.95	9.3 (8%)	27.4 (2%)	81.3 (7%)	264.1 (2%)	799.7 (7%)	2625.7 (2%)
	.99	1.3 (4%)	5.1 (1%)	6.2 (5%)	39.8 (1%)	51.2 (5%)	382.0 (1%)

If the first-stage sample size is increased, the advantage of the integrated approach increases: E.g., for *n*_1_=*n*_2_=9 and *Π*_0_=0.95, *Δ*=1, *α*_1_=0.5, the mean number of rejected hypotheses is 22% larger for the integrated approach than for the pilot approach.

### Correlated test statistics

Test statistics from genetic data are often stochastically dependent across hypotheses. In this section we study the impact of correlation between test statistics on the FDR and consider auto-correlation, block-correlation [12] and equi-correlation [16]. Auto-correlation may occur for example in microarray data because of spatial artefacts on the array or in gene association studies due to correlation between neighbouring markers. A block correlation structure, also called clumpy correlation, may be induced in microarray data for example by pathways of genes that are commonly regulated [17]. Finally, equi-correlation can be due to ‘array effects’ in microarray analyses.

For auto-correlation we consider an order among hypotheses and assume an autoregressive correlation structure. Here the correlation between the test statistics for hypotheses i and j is given by ^*ρ*|*i*−*j*|^. For block-correlation we assume that the test statistics are correlated in blocks of 20 hypotheses where the correlation between the test statistics within one block is *ρ*[12]. Hypotheses of different blocks are assumed to be independent. For equi-correlation we assume that for all pairs of hypotheses a pairwise correlation of *ρ*holds. For all correlation structures the alternatives are randomly distributed among the sequence of hypotheses. The simulations with correlated data were performed for the scenarios *m*∈{1000,10000,100000}, *m*_2_ ∈{0.01*m*,0.05*m*,0.1*m*}, *Δ*∈{1,1.6}), and *Π*_0_ ∈{0.95,0.99,1} with correlation coefficient *ρ*=0.5.

For block-correlation and auto-correlation the results are very similar to the independent case concerning the actual FDR. The mean number of rejected alternatives for the pilot and the integrated design are nearly identical (data not shown). For equi-correlated data the error rates of both selection procedures are maintained in all scenarios, even under the global null hypothesis. However, for most scenarios the procedure appears to be more conservative compared to the independent case. For scenarios with small *Δ* the mean number of rejected alternatives and the superiority of the integrated designs increases for the FDRS design (see Table 3). For the FNS design the differences between the integrated and the pilot design decrease (see Table 4). Note, however, that equi-correlation can be reduced or removed by adequate normalization [17].

**Table 3:** FDRS design for equi-correlated data

		***m=*1000**		***m=*10000**		***m=*100000**	
***α***_**1**_	***Π***_**0**_	***Δ=*1**	***Δ=*1.6**	***Δ=*1**	***Δ=*1.6**	***Δ=*1**	***Δ=*1.6**
0.1	.95	2.7 (3%)	18.1 (0%)	20.6 (5%)	169.9 (0%)	180.2 (5%)	1682.2 (0%)
	.99	0.4 (0%)	3.2 (0%)	2.0 (12%)	22.7 (0%)	16.4 (19%)	214.4 (1%)
0.2	.95	3.9 (9%)	21.5 (1%)	30.6 (12%)	203.4 (1%)	300.6 (14%)	2015.9 (1%)
	.99	0.6 (7%)	3.9 (1%)	2.9 (26%)	28.3 (1%)	26.0 (33%)	269.5 (2%)
0.5	.95	7.3 (22%)	26.8 (3%)	60.5 (28%)	257.3 (4%)	576.0 (29%)	2554.7 (4%)
	.99	1.1 (25%)	4.9 (3%)	5.7 (6%)	37.9 (4%)	48.8 (78%)	363.3 (4%)

**Table 4:** FNS design for equi-correlated data

		***m=*1000**		***m=*10000**		***m=*100000**	
***m***_**2**_	***Π***_**0**_	***Δ=*1**	***Δ=*1.6**	***Δ=*1**	***Δ=*1.6**	***Δ=*1**	***Δ=*1.6**
0.01*m*	.95	8.0 (13%)	15.2 (53%)	77.3 (13%)	141.2 (43%)	768.0 (13%)	1392.8 (41%)
	.99	2.4 (0%)	5.6 (1%)	17.7 (0%)	48.6 (1%)	168.5 (0%)	471.5 (1%)
0.05*m*	.95	14.4 (12%)	28.5 (4%)	135.4 (13%)	278.9 (4%)	1342.9 (13%)	2783.1 (4%)
	.99	3.0 (15%)	6.3 (3%)	21.6 (21%)	55.3 (4%)	207.9 (21%)	537.4 (5%)
0.1*m*	.95	15.8 (21%)	30.6 (5%)	149.0 (22%)	299.7 (5%)	1473.5 (22%)	2990.8 (5%)
	.99	3.2 (28%)	6.4 (5%)	22.8 (39%)	57.1 (8%)	216.4 (41%)	556.2 (8%)

### Real data application

We reanalysed the microarray data set by Tian [18]. In this experiment gene expression data were compared between 137 patients where bone lytic lesions could be detected by magnetic resonance imaging and 36 controls where such lesions could not be detected for 12625 probe sets. We used the pre-processed data set by Jeffery [19].

To obtain balanced group sizes for the re-analysis we arbitrarily selected 36 patients from the bone lytic lesions group. The samples were arbitrarily allocated to the two stages and the pilot and the integrated approach were applied for the FNS and the FDRS procedure and different parameters: *n*_1_={6,12} (*n*_2_=36−*n*_1_), *m*_2_={10,50,100,200}, *α*_1_={0.1,0.2,0.5,0.8}, *m*_*s*_=10. In the first stage for all procedures a two-sided *t*-test was computed. For the integrated procedures we computed sequential *p*-values based on (2) using a normal approximation where the critical values from the model with known variances are applied to the *p*-values of the *t*-test.

Table 5 shows that in most scenarios the integrated procedure rejects more hypotheses than the corresponding pilot procedure for both selection rules. This difference is larger for larger first stage sample sizes and for increasing parameters *m*_2_ or *α*_1_, respectively. Only for small *α*_1_ and *n*_1_=6 the integrated and the pilot approach of the FDRS procedure reject approximately the same number of hypotheses. Note that no hypothesis was significant at the final test decision which was not considered in the second stage. Setting *m*_*s*_=0 the results for the integrated FDRS procedure did not change.

**Table 5:** Real data application

	**FNS**		**FDRS**		
***n***_**1**_**/*n***_**2**_	***m***_**2**_	**Rejections**	***α***_**1**_	**Rejections**	**m**_**2**_^**FDRS**^
6 / 30	10	6 (1)	0.1	0 (0)	1
	50	15 (10)	0.2	1 (1)	2
	100	30 (12)	0.5	28 (21)	85
	200	68 (30)	0.8	345 (132)	2291
12 / 24	10	8 (4)	0.1	3 (3)	3
	50	33 (8)	0.2	51 (38)	84
	100	60 (17)	0.5	398 (150)	1745
	200	109 (37)	0.8	573 (99)	5887

## Discussion and conclusion

In this paper we discussed several selection rules for two-stage designs, where after an interim analysis only promising hypotheses are considered in the second stage.

For the choice of the selection rule, different criteria may apply. With the FNS design, the total number of observations is known in advance, which facilitates the planning of resources. However, this design does not adapt to the number of hypotheses that show an effect in the interim analysis. The latter can be achieved with the FDRS design, where, on the other hand, the total number of observations is random and the planning of resources becomes more difficult. As an extension one can consider an FDRS design where the overall number of observations (across all hypotheses and both stages) is fixed and the observations allocated to the second stage are equally distributed among the selected hypotheses. This comes at the cost of a decreasing per hypothesis power if for a larger number of hypotheses the alternative holds.

For the FNS design the testing procedures provided a sound control in the considered scenarios where more than 5 hypotheses are selected for the second stage for independent as well as for correlated data. Also for the modified FDRS procedure FDR control is given in all scenarios for *m*_*s*_ > 5. Comparing the integrated approaches for both selection rules with the corresponding pilot approaches showed an advantage of the integrated approach in many scenarios. This holds particularly for the FNS design but in many scenarios also for the FDRS design. The advantage of the integrated design increases with the proportion of observations allocated to the first stage. This is in line with earlier findings [7,8], where scenarios with small first-stage sample sizes were considered and only small differences between the integrated and the pilot design have been observed. In particular, if the effect sizes in microarray studies are low (as, e.g., shown in examples in [20]) and the number of observations in the first stage is sufficiently large compared to the number of observations for the second stage, the integrated design is superior.

On the other hand, using only the second-stage data for testing has the advantage of increased flexibility and simplicity. For example, the pilot FNS procedure controls the FDR even if the hypotheses for the second stage are selected in an arbitrary way. Furthermore, standard (non-sequential) tests can be applied and FDR control can be shown analytically under suitable assumptions.

In the simulations the BH-procedure was applied to the sequential *p*-values to control the FDR. As described above, this method is conservative if *Π*_0_ < 1 as it controls the FDR actually at level *Π*_0_*α*. Following the suggestion of one of the referees, we additionally considered so called adaptive FDR controlling procedures that are based on an estimate of *Π*_0_(see Additional file 2). Under independence these adaptive tests are less conservative then the BH-tests, but did not exceed the nominal level in the considered simulation scenarios. However, as shown earlier (e.g., [21]) under strong correlation adaptive procedures may inflate the FDR.

It is well known that two-stage designs may lead to a considerable improvement in efficiency compared to single-stage designs [1-8] and this applies also to the procedures investigated in this paper (see Additional file 3 for a simulation study comparing the two-stage tests to corresponding single-stage designs). Furthermore, the methods can be extended to designs where an explicit early rejection boundary is applied in the interim analysis as in many group-sequential applications. In this case the calculation of the sequential *p*-values is slightly modified (the integral boundaries depend on the early rejection boundaries). However, unless the fraction of hypotheses for which the alternative holds is large, it is expected that the addition of an early rejection boundary at the interim analysis has only a marginal impact on the efficiency of the procedure. Furthermore, for hypotheses that are rejected in the interim analysis based on few observations, a confirmation with a larger sample size might be important anyway.

## Appendix

### Asymptotic considerations

In this section we argue that asymptotically, for increasing number of hypotheses, the FNS and the FDRS selection rule are equivalent to a selection rule where hypotheses are selected based on a fixed threshold *γ*_1_. Let $R(\gamma )=♯\{p_{i}^{\left(1\right)}\leq \gamma \}$ denote the number of hypotheses with a first-stage *p*-value not exceeding *γ*, and *V*(*γ*)(*S*(*γ*)) the respective number of *p*-values corresponding to true null (alternative) hypotheses, respectively. Let *m*_0_ and *m*_1_ denote the number of true null and alternative hypotheses, respectively. Consider the following assumptions: 

The empirical distribution functions of the first-stage *p*-values corresponding to the null and the alternative hypotheses converge almost surely, i.e., $\underset{m\to \infty }{\lim}V(\gamma )/m_{0}=\gamma$ and $\underset{m\to \infty }{\lim}S(\gamma )/m_{1}=F_{1}(\gamma )$ exist for all *γ*∈(0,1], where *F*_1_ is a continuous strictly increasing function.

$\underset{m\to \infty }{\lim}m_{0}/m=\Pi_{0}^{\infty }$ exists.

For the FNS procedure assume that $\underset{m\to \infty }{\lim}$*m*_2_/*m*=*δ*exists and let *G*(*p*)=*Π*_0_*p* + (1−*Π*_0_)*F*_1_(*p*) denote the limiting distribution function of the first-stage *p*-values. By the continuity and strict monotonicity of *G* there is a unique $\gamma_{1}^{\infty }$ such that $G(\gamma_{1}^{\infty })=\delta$. Now, $\underset{m\to \infty }{\lim}p_{\left(m_{2}\right)}=\gamma_{1}^{\infty }$ almost surely. If we additionally assume that for all finite *m* the distribution of a (randomly chosen) *p*-value is given by *G*(*p*) with density *g*, then *γ*_1_ is the *m*_2_/*m* quantile of *G*. Thus, its variance is given by $\left(m_{2}/m)(1-m_{2}/m)/(\mathrm{mg}{\left(m_{2}/m\right)}^{2}\right)$[22].

For the FDRS procedure *γ*_1_=*m*_2_/*mα* corresponds to the critical value that results from the BH-procedure. If (1) and (2) hold and *Π*_0_<1, it follows as in [12] (Theorem 5) that this critical value converges almost surely.

### Computation of the two-sided sequential *p*-value

If the hypothesis _*Hi*_,*i*=1,…,*m*is selected for the second stage (i.e., if the first-stage *p*-value is smaller than *γ*_1_), the two-sided sequential *p*-value given by (2) is calculated by numerical integration:

(3) $$\begin{array}{lll} p_{i}= & \;\overset{\infty }{\underset{c_{1-\gamma_{1}/2}}{\int }}\left[1-\text{Φ}(\frac{|z_{i}|-a}{b})\right]\varphi (z)\;\mathrm{dz}\; & \;\\ & +\overset{\infty }{\underset{c_{1-\gamma_{1}/2}}{\int }}\left[\text{Φ}(\frac{-|z_{i}|-a}{b})\right]\varphi (z)\;\mathrm{dz}\;\\ & +\overset{c_{\gamma_{1}/2}}{\underset{-\infty }{\int }}\left[1-\text{Φ}(\frac{|z_{i}|-a}{b})\right]\varphi (z)\;\mathrm{dz}\; & \;\\ & +\overset{c_{\gamma_{1}/2}}{\underset{-\infty }{\int }}\left[\text{Φ}(\frac{-|z_{i}|-a}{b})\right]\varphi (z)\;\mathrm{dz}\; & \; \end{array}$$

with $a=\sqrt{\frac{n_{1}}{n_{1}+n_{2}}}\;z$ and $b=\sqrt{\frac{n_{2}}{n_{1}+n_{2}}}$. Here *φ*(.), Φ(.), and $c_{1-\gamma_{1}/2}$ denote the density, the cumulative distribution function, and the (1−*γ*_1_/2)-quantile of the standard normal distribution, respectively. If the first-stage *p*-value is larger than *γ*_1_, $p_{i}=p_{i}^{\left(1\right)}$.

## Competing interests

Both authors have no competing interests.

## Author’s contributions

Both authors contributed equally to the development of the methods, the design of the simulations, and to writing the paper. SZ conducted the simulations and data analyses. All authors read and approved the final manuscript.

## Authors’ information

The views expressed are those of the author (MP) and should not be understood or quoted as being made on behalf of the European Medicines Agency or its scientific Committees.

## Supplementary Material

Additional file 1 **We report the simulation scenarios and results of the simulation study assessing the FDR of the FNS and FDRS design (modified procedure with m_s_=6) for the case of independent test statistics as described in the results section of the manuscript.** For each scenario at least 1000 simulation runs were performed. For scenarios with lower *m* the simulation runs were increased to 50000 (*m*={100;500}), 20000 (*m*=1000), and 10000 (*m*=5000), because in these scenarios there is a higher variability of the false discovery proportion such that the estimator of the FDR converges slower. This also holds if *m* is large but *Π*_0_ ≈ 1 or *Δ* is small. Therefore, for these scenarios the number of simulation runs was increased. The resulting FDR values were plotted as a function of *α*_1_ for the FDRS design (left column) or as a function of *m*_2_ for the FNS design (right column), respectively. Click here for file

Additional file 2 Results of a simulation study for two-stage designs where an adaptive test procedures is applied based on an estimator for the proportion of true null hypotheses. Click here for file

Additional file 3 Two single-stage designs are compared to the results: For the first single-stage design the sample size for each hypothesis is n_1_, for the second design the sample size is n_1_ + n_2_. For the first design we compare the gain in power of the integrated design and for the second design the attention lies on the reduction in costs. Click here for file
