# Supplementary material for: False discovery rate control in two-stage designs
Source: BMC Bioinformatics. 2012 May 6;13:81. doi: 10.1186/1471-2105-13-81 (PMC3496575; doi:10.1186/1471-2105-13-81)
Supplement: Additional file 1 — We report the simulation scenarios and results of the simulation study assessing the FDR of the FNS and FDRS design (modified procedure with ms=6) for the case of independent test statistics as described in the results section of the manuscript. For each scenario at least 1000 simulation runs were performed. For scenarios with lower m the simulation runs were increased to 50000 (m={100;500}), 20000 (m=1000), and 10000 (m=5000), because in these scenarios there is a higher variability of the false discovery proportion such that the estimator of the FDR converges slower. This also holds if m is large but Π0 ≈ 1 or Δ is small. Therefore, for these scenarios the number of simulation runs was increased. The resulting FDR values were plotted as a function of α1 for the FDRS design (left column) or as a function of m2 for the FNS design (right column), respectively. [file 1471-2105-13-81-S1.pdf]

Actual FDR values for the FNS and the FDRS design

We investigate the actual FDR of the proposed testing procedures for the FNS and FDRS selection rules for the case of independent test statistics as described in the Results section of the manuscript. We consider the one-sample z-test for m two-sided null hypotheses H_{0i}: $\mu_i = 0$ versus H_{1i}: $\mu_i \neq 0$, $i = 1, \dots, m$, for the mean of normally distributed observations with $n_1 = 6$, $n_2 = 12$ and nominal significance level $\alpha = 0.05$. Note that we present results for the modified FDRS procedure with $m_s = 6$.

The considered scenarios are those described in the table below. For each scenario at least 1000 simulation runs were performed. For scenarios with lower m the simulation runs were increased to 50000 ($m = \{100; 500\}$), 20000 ($m = 1000$), and 10000 ($m = 5000$), because in these scenarios there is a higher variability of the false discovery proportion such that the estimator of the FDR converges slower. This also holds if m is large but $\pi_0 \approx 1$ or Δ is small. Therefore, for these scenarios the number of simulation runs was increased. In the following pages the resulting FDR values were plotted as a function of α_1 for the FDRS design (left column) or as a function of m_2 for the FNS design (right column), respectively.

Table - Parameter values

Simulations were performed for all combinations of parameter values listed in the table. For the alternative hypotheses we assume that the data are $N(\delta_i, 1)$ distributed where the δ_i are alternating $\{\pm\Delta, \pm 3\Delta/4, \pm\Delta/2, \pm\Delta/4\}$, and the values Δ are as given in the table. Furthermore, results for $m \in \{10000, 100000\}$ for the scenarios $m_2 \in \{0.01m, 0.05m, 0.1m\}$ with $\Delta \in \{1, 1.6\}$), and $\pi_0 \in \{0.95, 0.99, 1\}$ are reported in the manuscript.

Parameter	Values
m	$100, 500, 1000, 5000, 10000, 100000$
π_0	$.5, .6, \dots, .9, .91, \dots, 1$
Δ	$.2, .4, \dots, 2$
α_1	$0.01, 0.02, \dots, 0.4, 0.42, \dots, 0.5$
m_2	1, 2, ..., 20, 22, ..., 50, 55, ..., 100

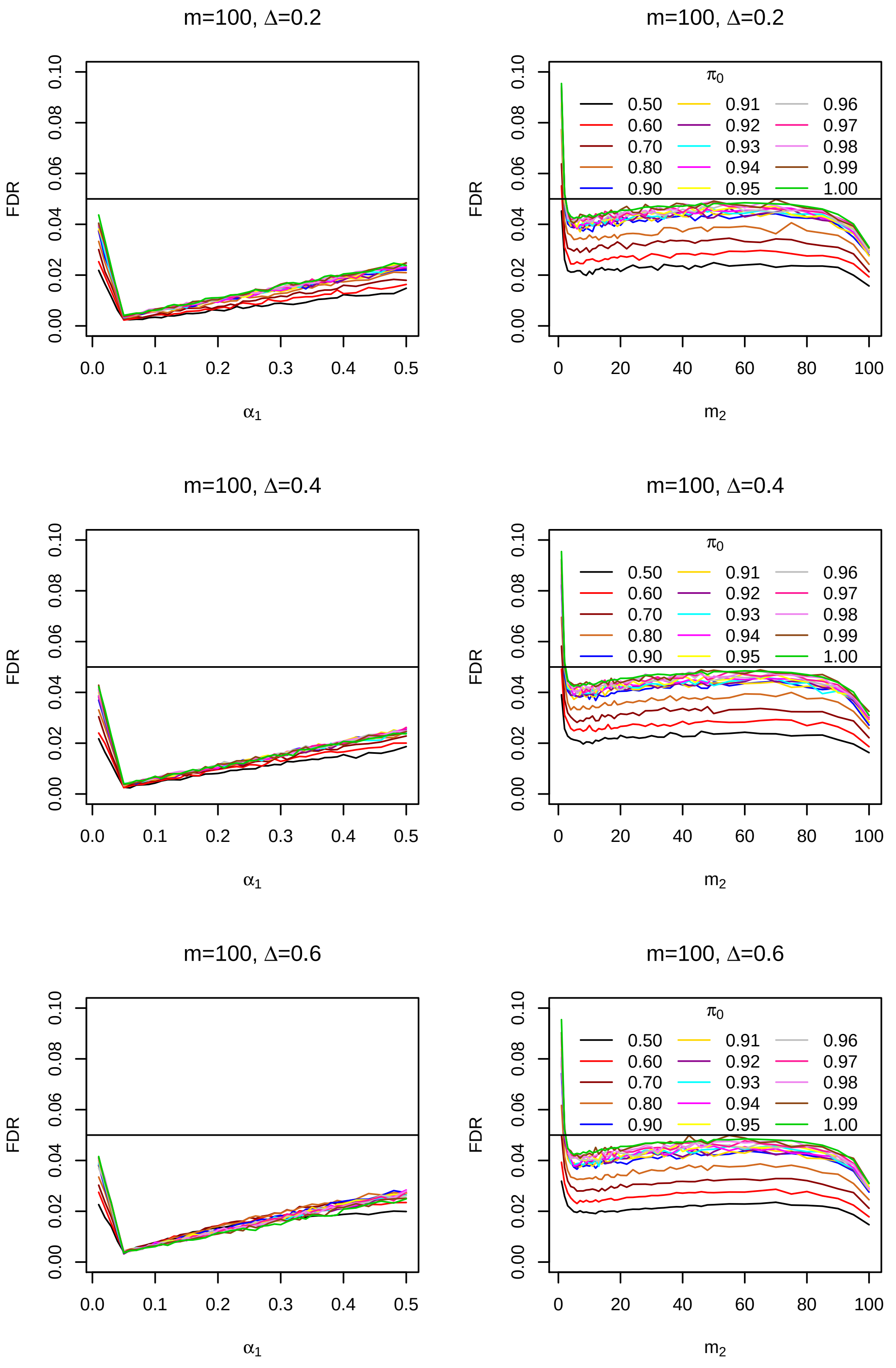
m=100, Δ=0.2
m=100, Δ=0.2
m=100, Δ=0.4
m=100, Δ=0.4
m=100, Δ=0.6
m=100, Δ=0.6
FDR
α1
m2
π0
0.50
0.60
0.70
0.80
0.90
0.91
0.92
0.93
0.94
0.95
0.96
0.97
0.98
0.99
1.00
0.00
0.02
0.04
0.06
0.08
0.10
0.0
0.1
0.2
0.3
0.4
0.5
0
20
40
60
80
100

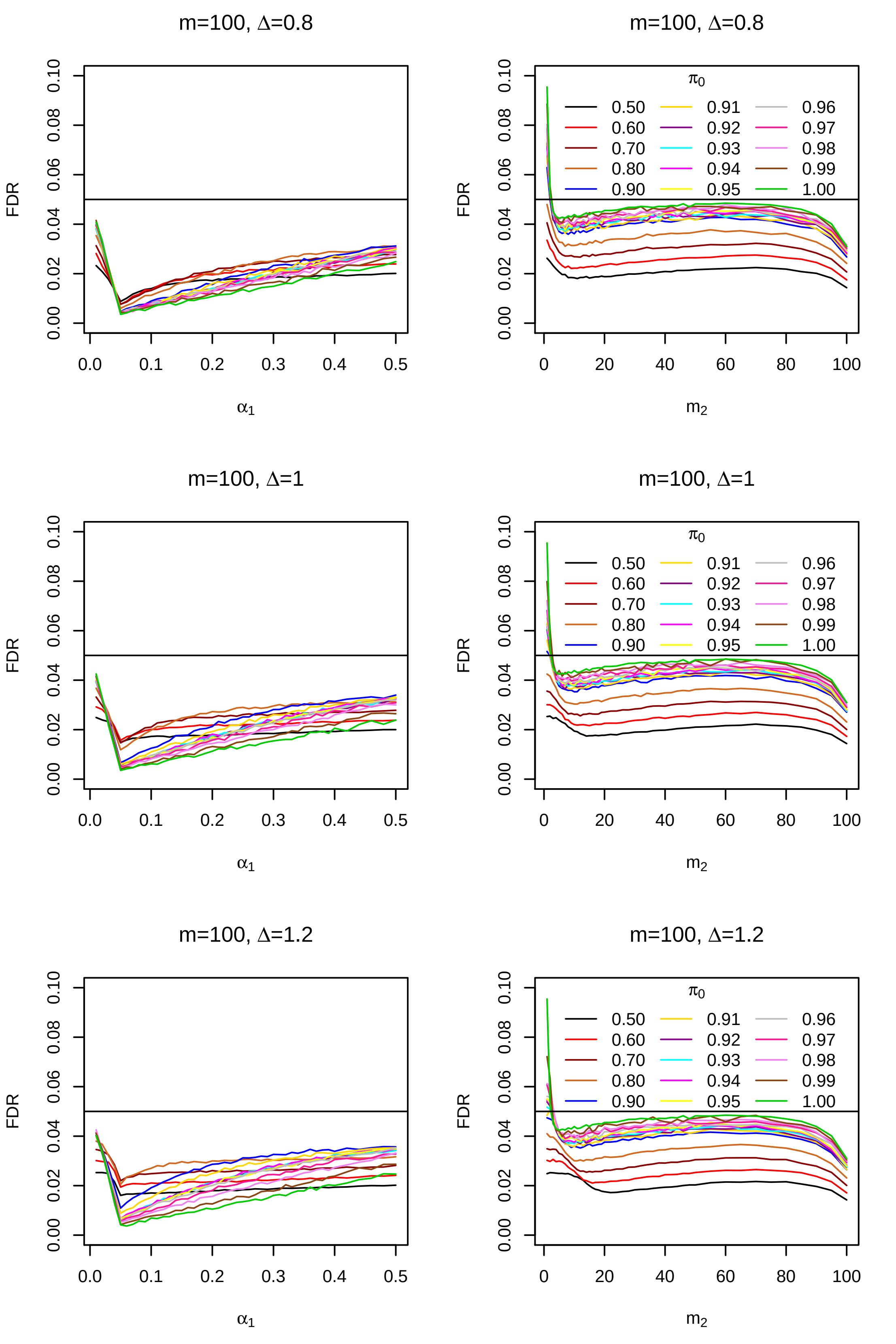
m=100, Δ=0.8
FDR
0.00
0.02
0.04
0.06
0.08
0.10
0.0
0.1
0.2
0.3
0.4
0.5
α1
m=100, Δ=0.8
π0
0.50
0.60
0.70
0.80
0.90
0.91
0.92
0.93
0.94
0.95
0.96
0.97
0.98
0.99
1.00
0
20
40
60
80
100
m2
m=100, Δ=1
α1
m=100, Δ=1
m2
m=100, Δ=1.2
α1
m=100, Δ=1.2
m2

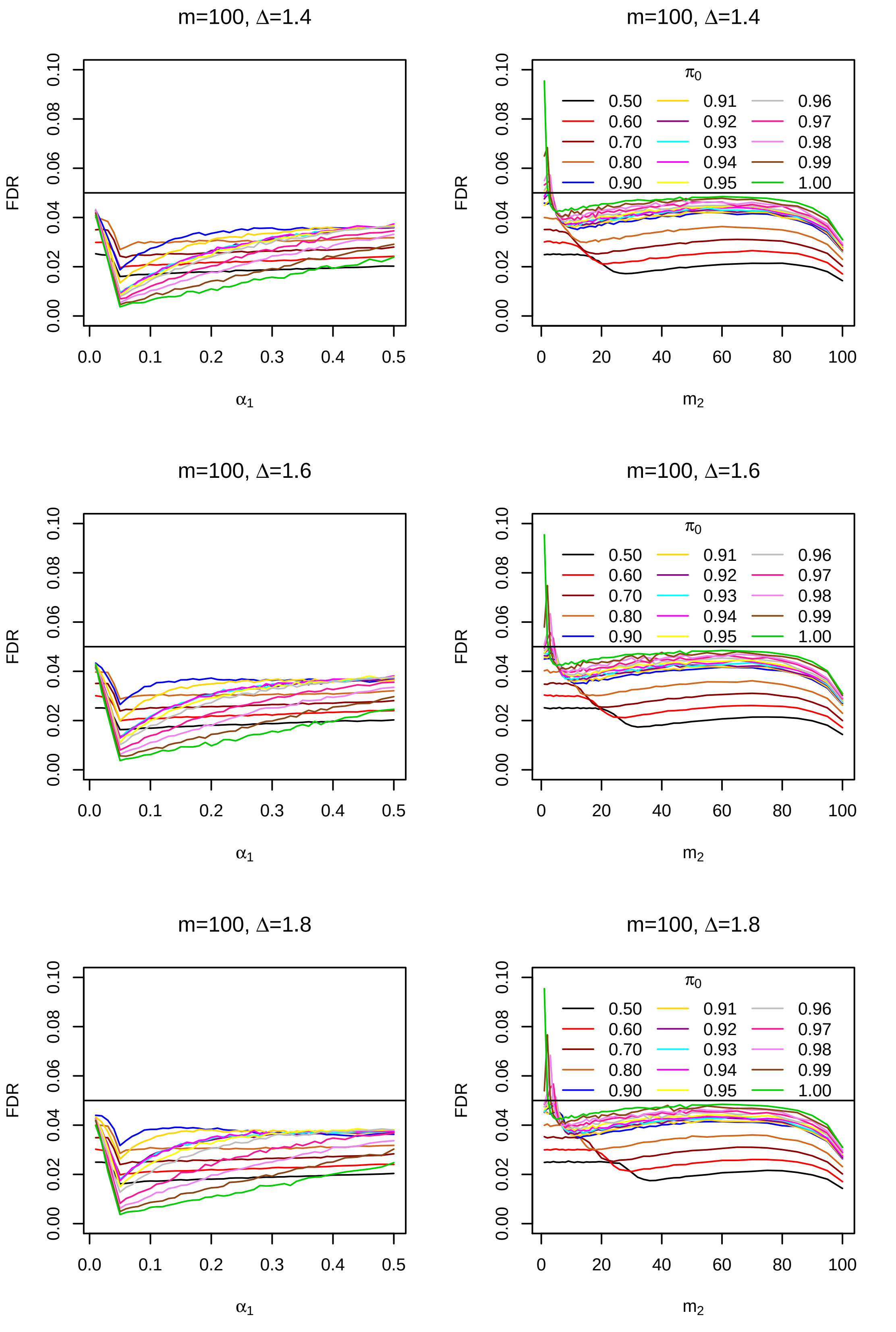

m=100, Δ=1.4
m=100, Δ=1.4
m=100, Δ=1.6
m=100, Δ=1.6
m=100, Δ=1.8
m=100, Δ=1.8
FDR
α1
m2
π0
0.50 0.60 0.70 0.80 0.90
0.91 0.92 0.93 0.94 0.95
0.96 0.97 0.98 0.99 1.00

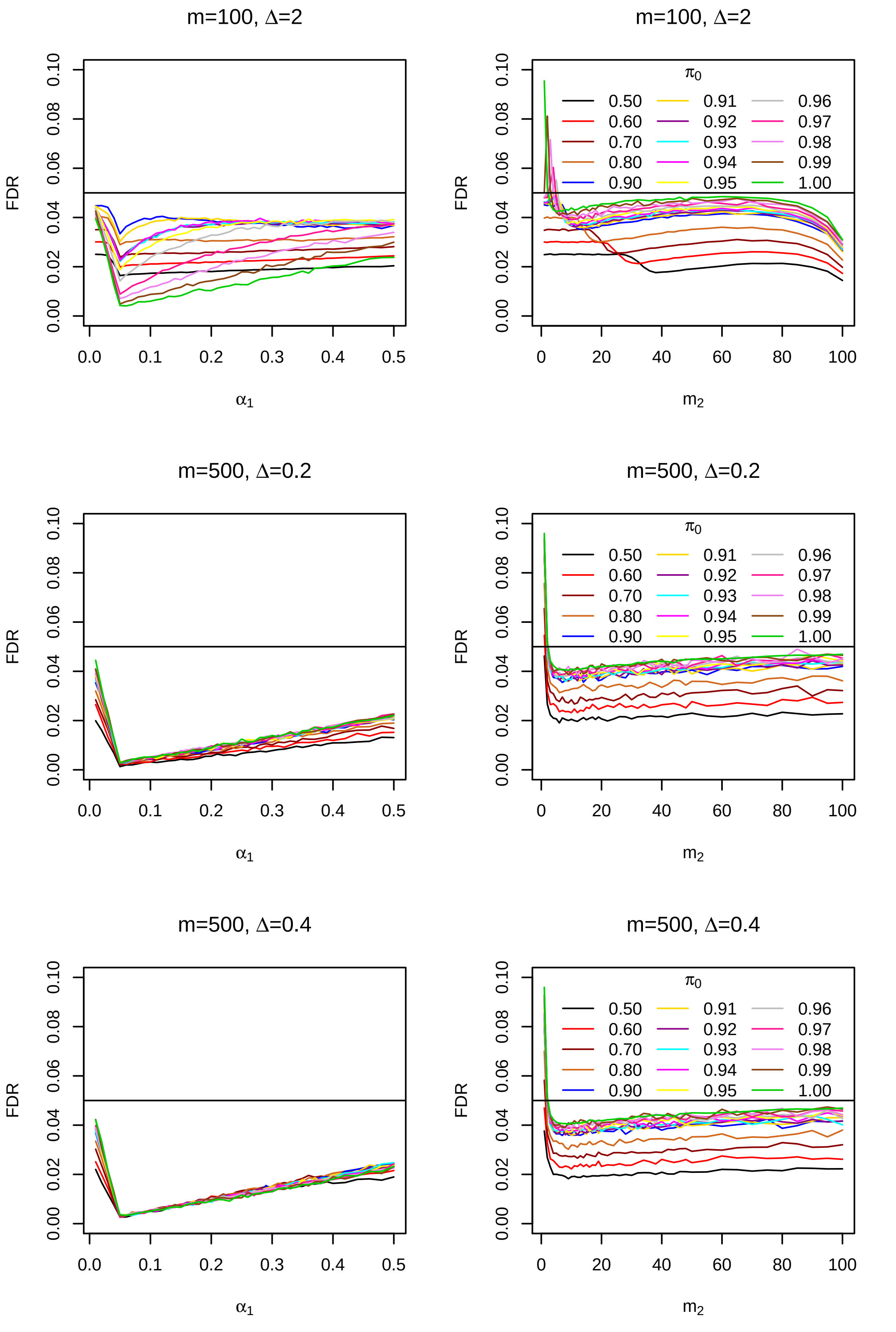

m=100, Δ=2
m=500, Δ=0.2
m=500, Δ=0.4
FDR
α1
m2
π0
0.50
0.60
0.70
0.80
0.90
0.91
0.92
0.93
0.94
0.95
0.96
0.97
0.98
0.99
1.00

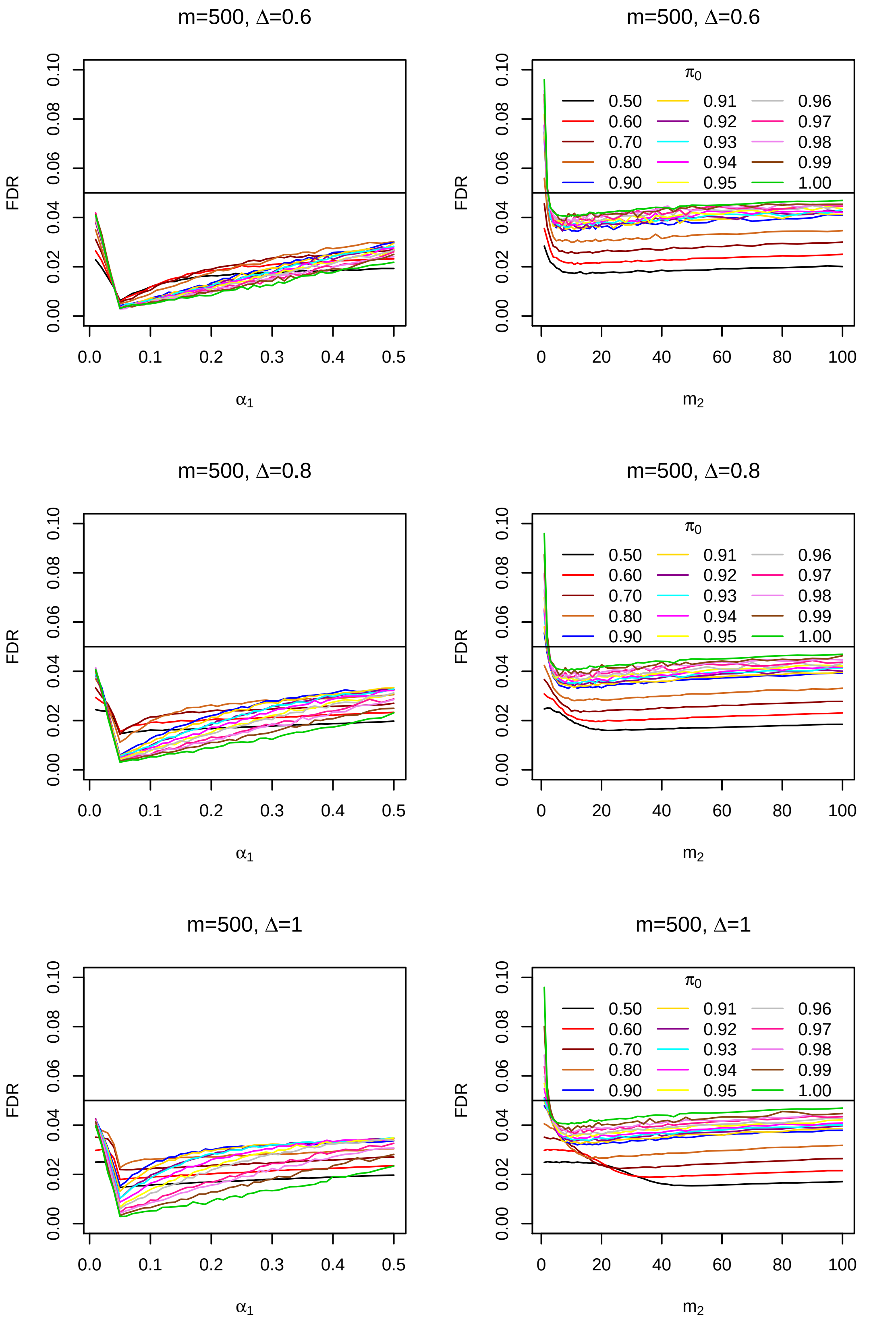

m=500, Δ=0.6
FDR
α1
m2
π0
0.50
0.60
0.70
0.80
0.90
0.91
0.92
0.93
0.94
0.95
0.96
0.97
0.98
0.99
1.00
m=500, Δ=0.8
m=500, Δ=1

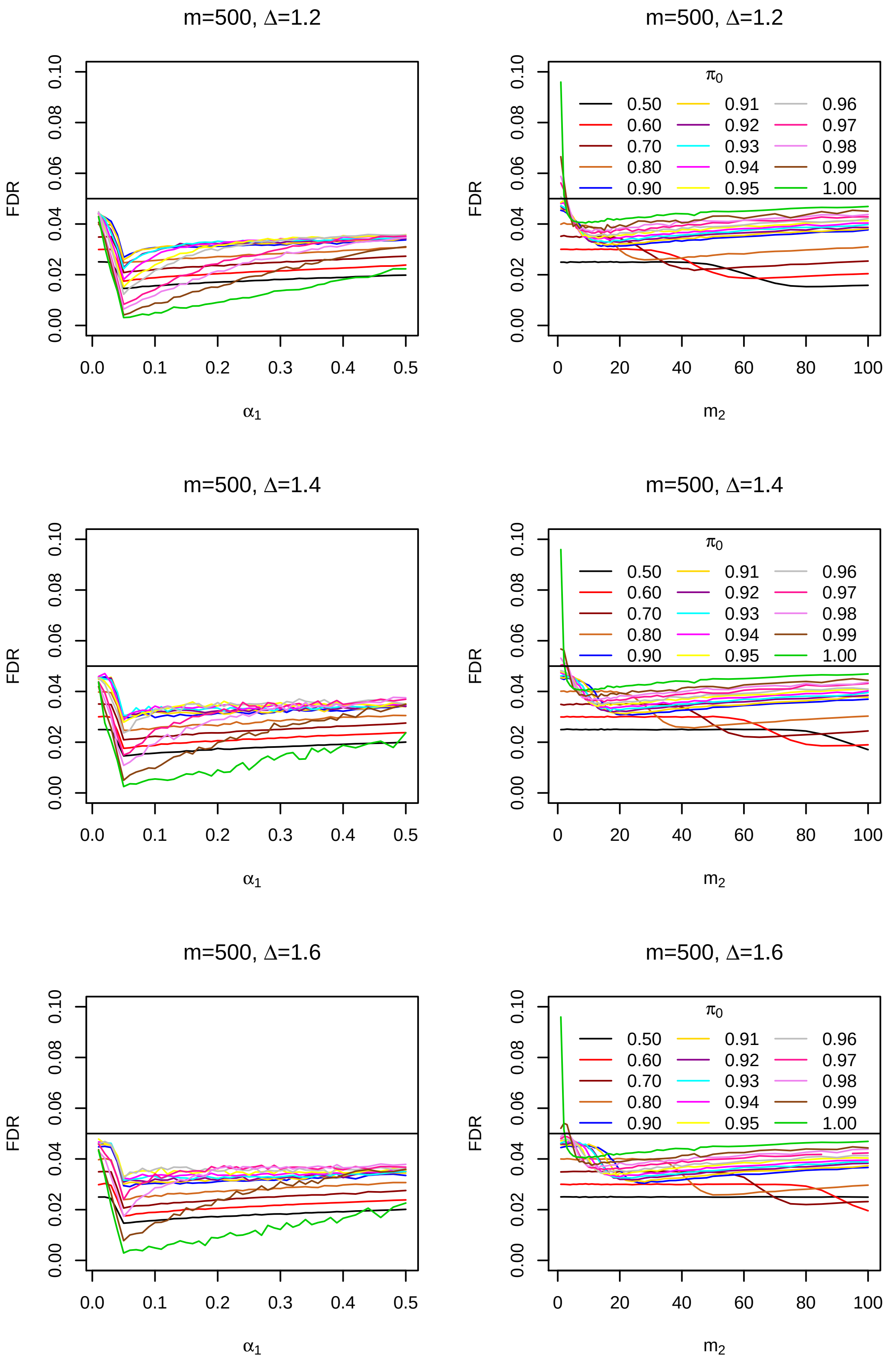

m=500, Δ=1.2
m=500, Δ=1.4
m=500, Δ=1.6
FDR
α_1
m_2
π_0
0.50
0.60
0.70
0.80
0.90
0.91
0.92
0.93
0.94
0.95
0.96
0.97
0.98
0.99
1.00
0.00 0.02 0.04 0.06 0.08 0.10
0.0 0.1 0.2 0.3 0.4 0.5
0 20 40 60 80 100

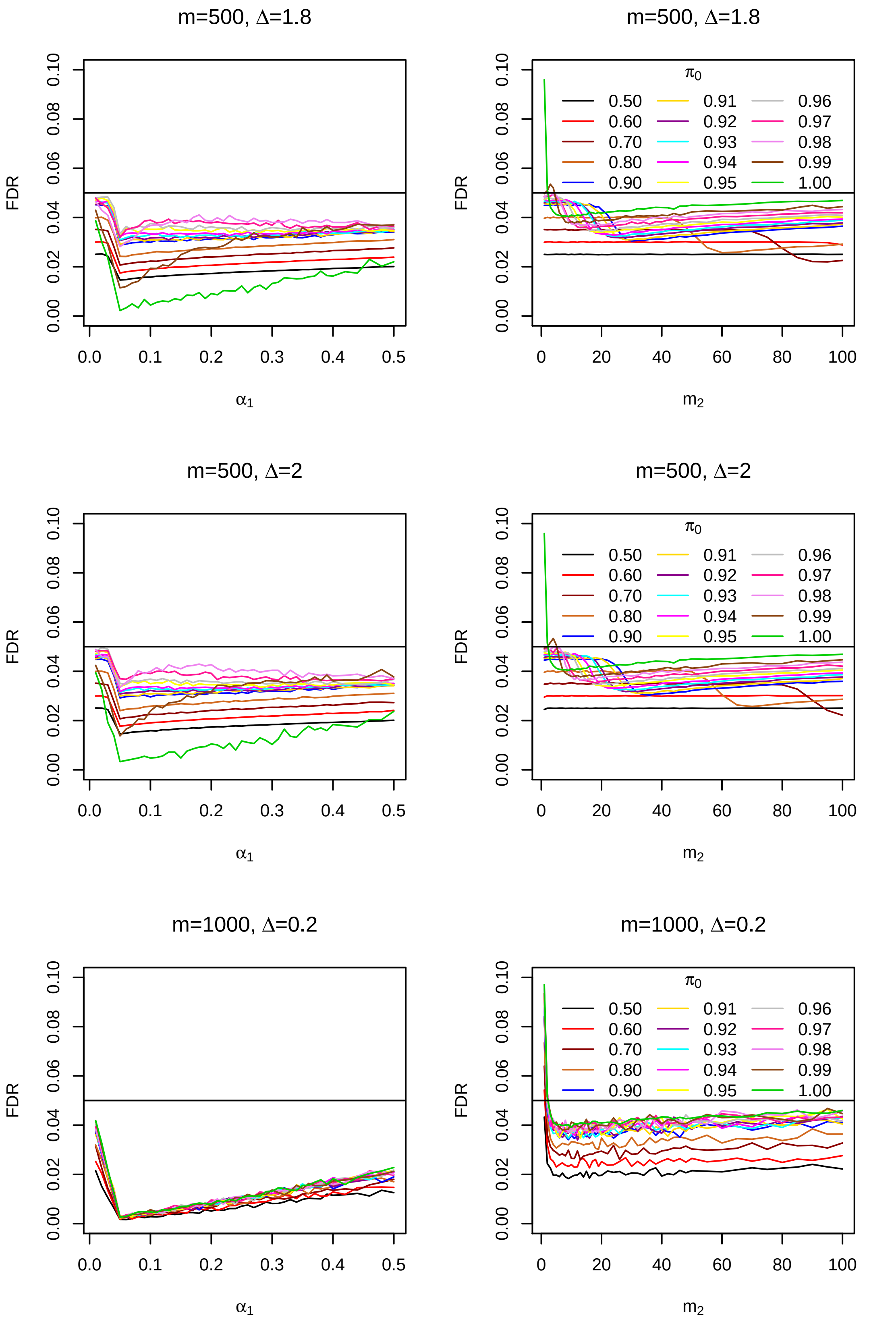

m=500, Δ=1.8
m=500, Δ=2
m=1000, Δ=0.2
FDR
α1
m2
π0
0.50 0.60 0.70 0.80 0.90 0.91 0.92 0.93 0.94 0.95 0.96 0.97 0.98 0.99 1.00
0.00 0.02 0.04 0.06 0.08 0.10
0.0 0.1 0.2 0.3 0.4 0.5
0 20 40 60 80 100

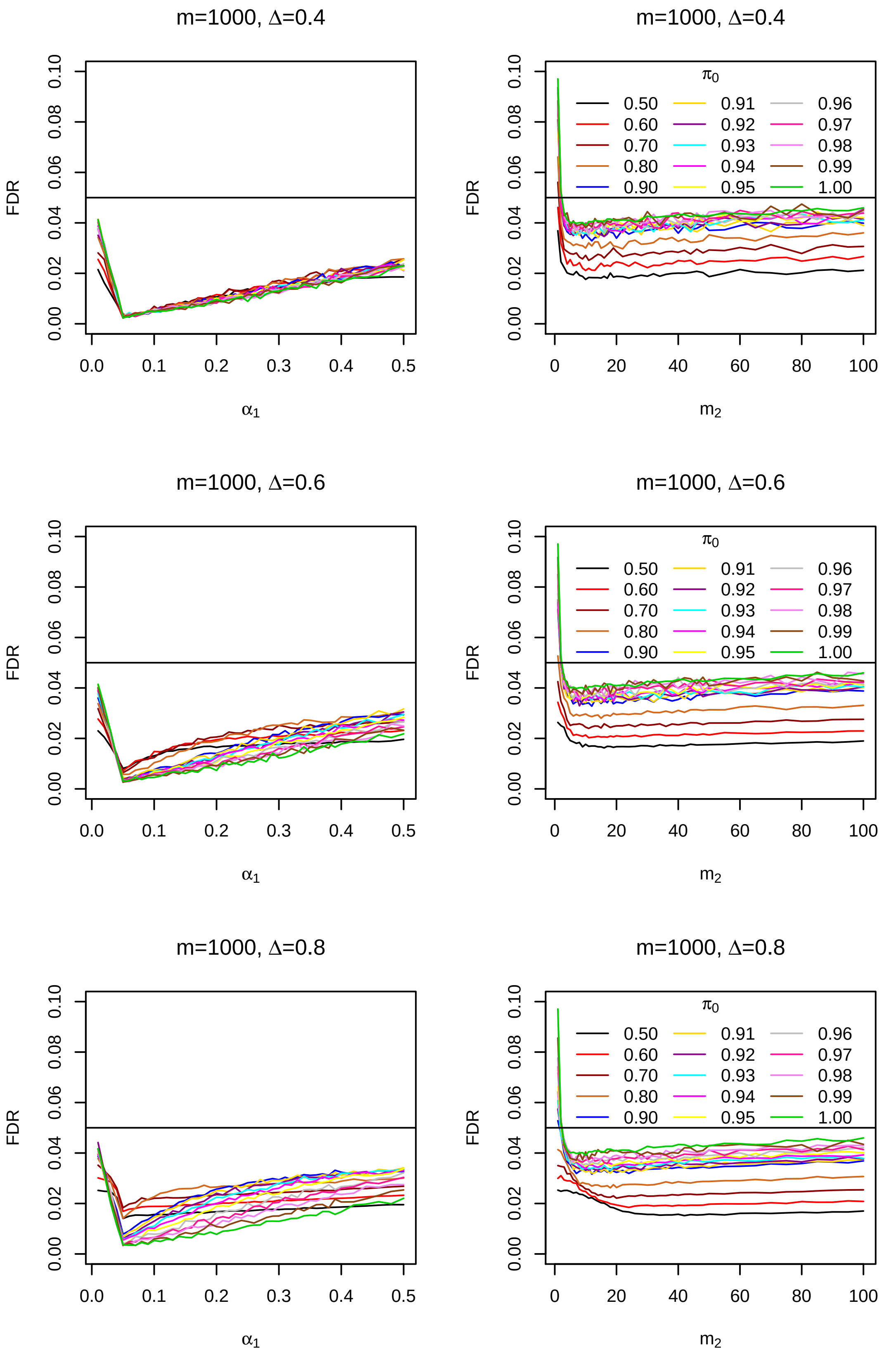

m=1000, Δ=0.4
FDR
0.00
0.02
0.04
0.06
0.08
0.10
0.0
0.1
0.2
0.3
0.4
0.5
α1
m=1000, Δ=0.4
FDR
π0
0.50
0.60
0.70
0.80
0.90
0.91
0.92
0.93
0.94
0.95
0.96
0.97
0.98
0.99
1.00
0
20
40
60
80
100
m2
m=1000, Δ=0.6
FDR
α1
m=1000, Δ=0.6
FDR
π0
m2
m=1000, Δ=0.8
FDR
α1
m=1000, Δ=0.8
FDR
π0
m2

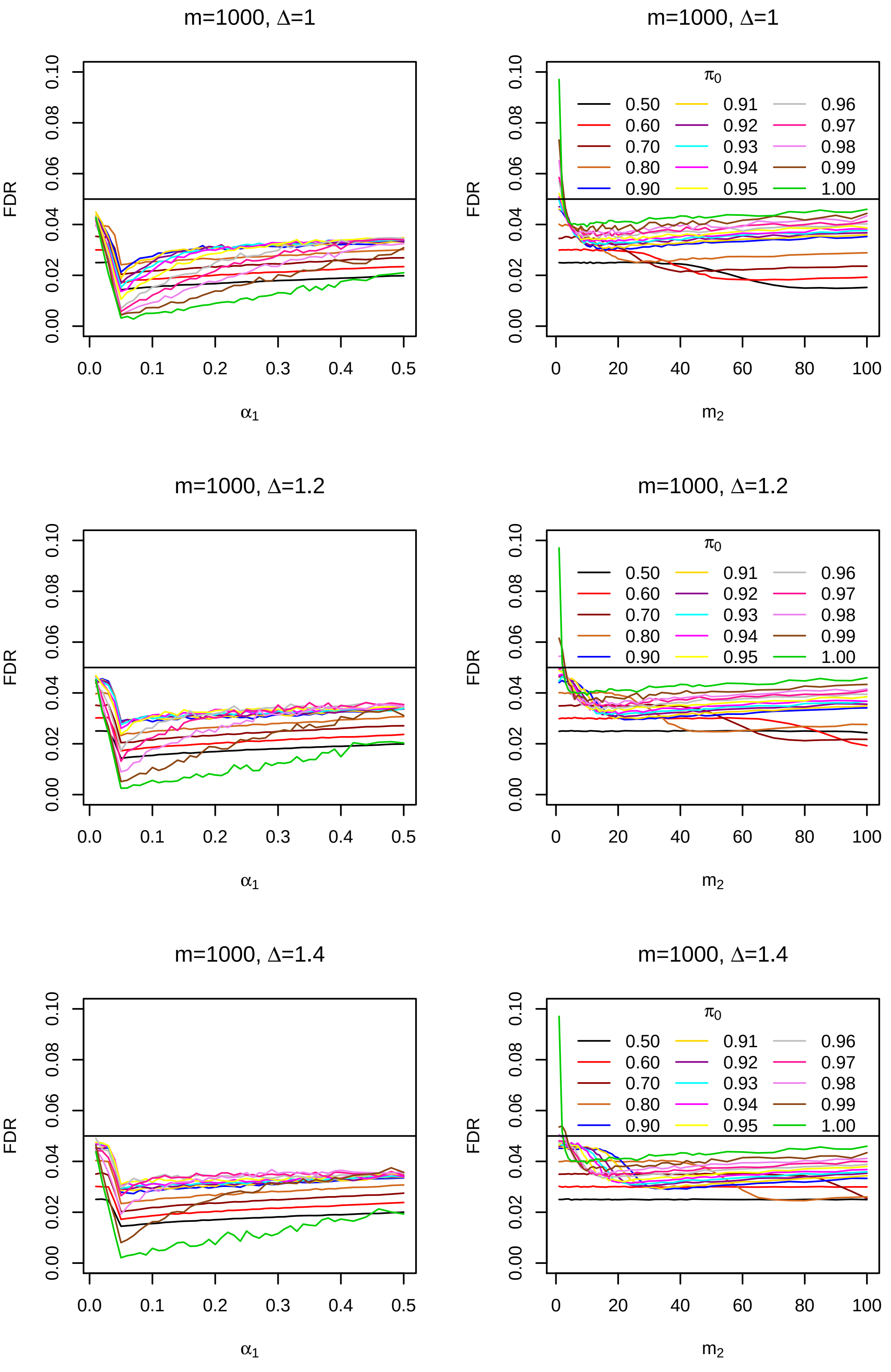

m=1000, Δ=1
m=1000, Δ=1
m=1000, Δ=1.2
m=1000, Δ=1.2
m=1000, Δ=1.4
m=1000, Δ=1.4
FDR
0.00
0.02
0.04
0.06
0.08
0.10
0.0
0.1
0.2
0.3
0.4
0.5
α_1
0
20
40
60
80
100
m_2
π_0
0.50
0.60
0.70
0.80
0.90
0.91
0.92
0.93
0.94
0.95
0.96
0.97
0.98
0.99
1.00

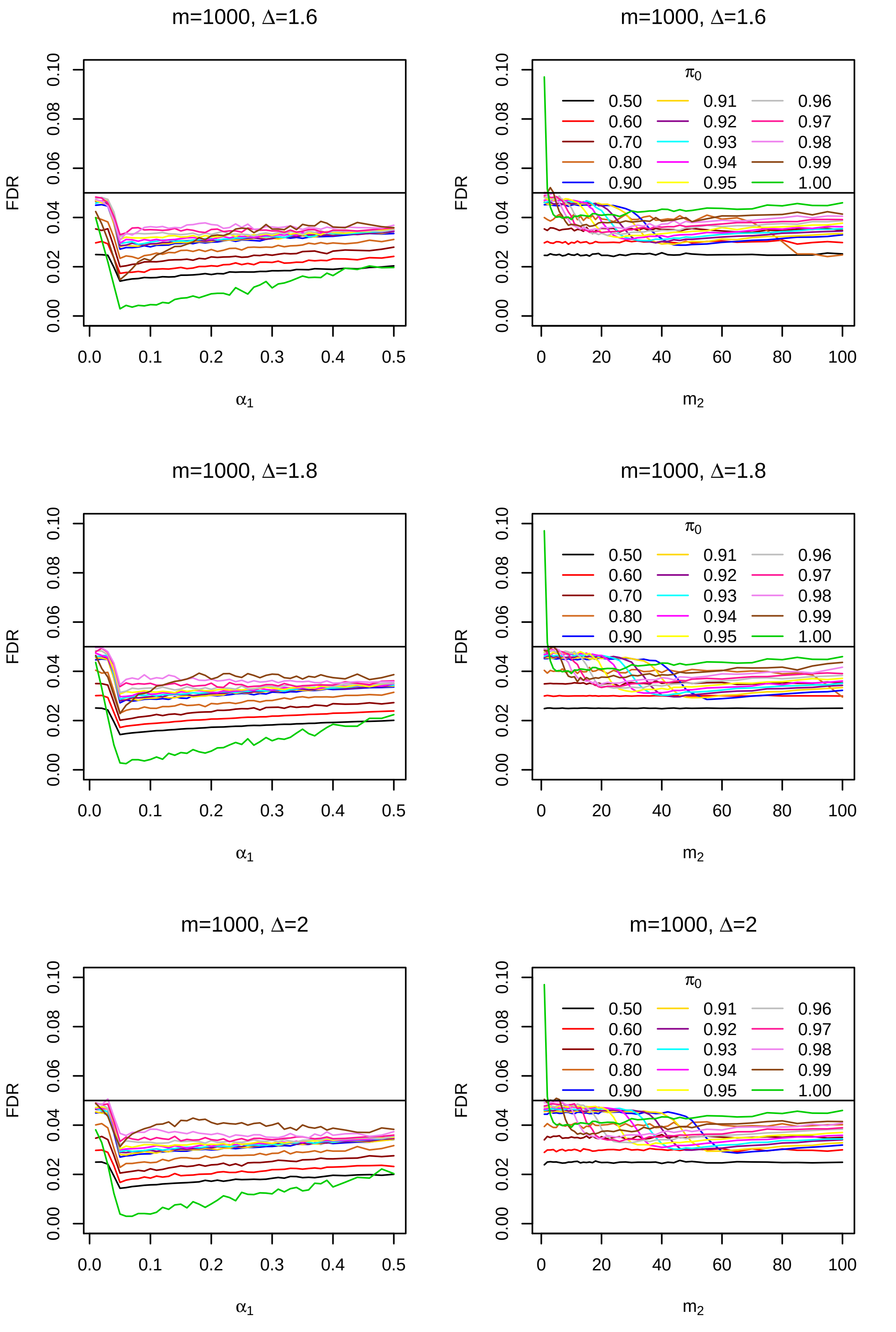

m=1000, Δ=1.6
FDR
α1
m=1000, Δ=1.6
FDR
m2
π0
0.50 0.60 0.70 0.80 0.90
0.91 0.92 0.93 0.94 0.95
0.96 0.97 0.98 0.99 1.00
m=1000, Δ=1.8
FDR
α1
m=1000, Δ=1.8
FDR
m2
π0
0.50 0.60 0.70 0.80 0.90
0.91 0.92 0.93 0.94 0.95
0.96 0.97 0.98 0.99 1.00
m=1000, Δ=2
FDR
α1
m=1000, Δ=2
FDR
m2
π0
0.50 0.60 0.70 0.80 0.90
0.91 0.92 0.93 0.94 0.95
0.96 0.97 0.98 0.99 1.00

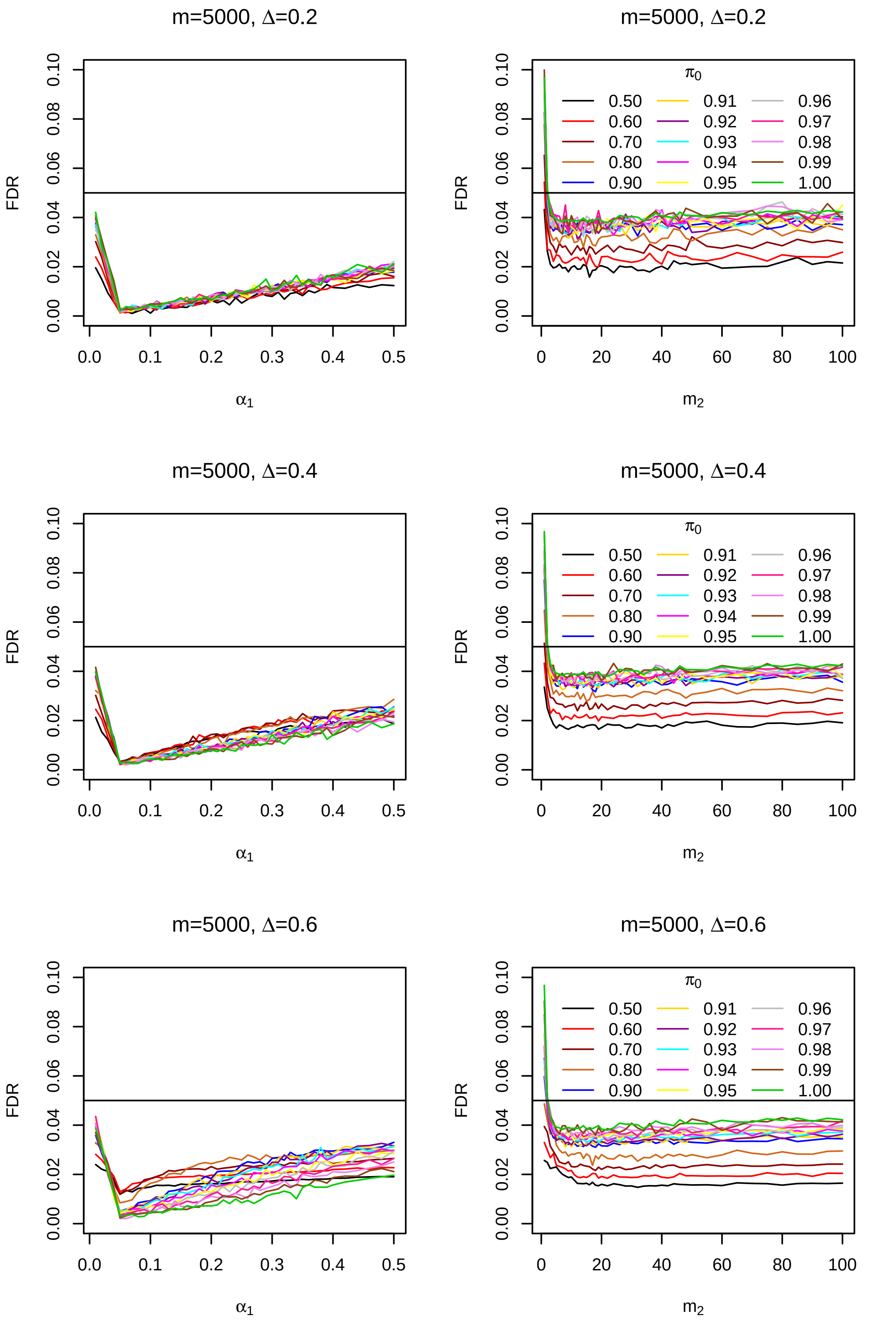

m=5000, Δ=0.2
FDR
0.00
0.02
0.04
0.06
0.08
0.10
0.0
0.1
0.2
0.3
0.4
0.5
α1
m=5000, Δ=0.2
FDR
π0
0.50
0.60
0.70
0.80
0.90
0.91
0.92
0.93
0.94
0.95
0.96
0.97
0.98
0.99
1.00
0
20
40
60
80
100
m2
m=5000, Δ=0.4
FDR
α1
m=5000, Δ=0.4
FDR
π0
m2
m=5000, Δ=0.6
FDR
α1
m=5000, Δ=0.6
FDR
π0
m2

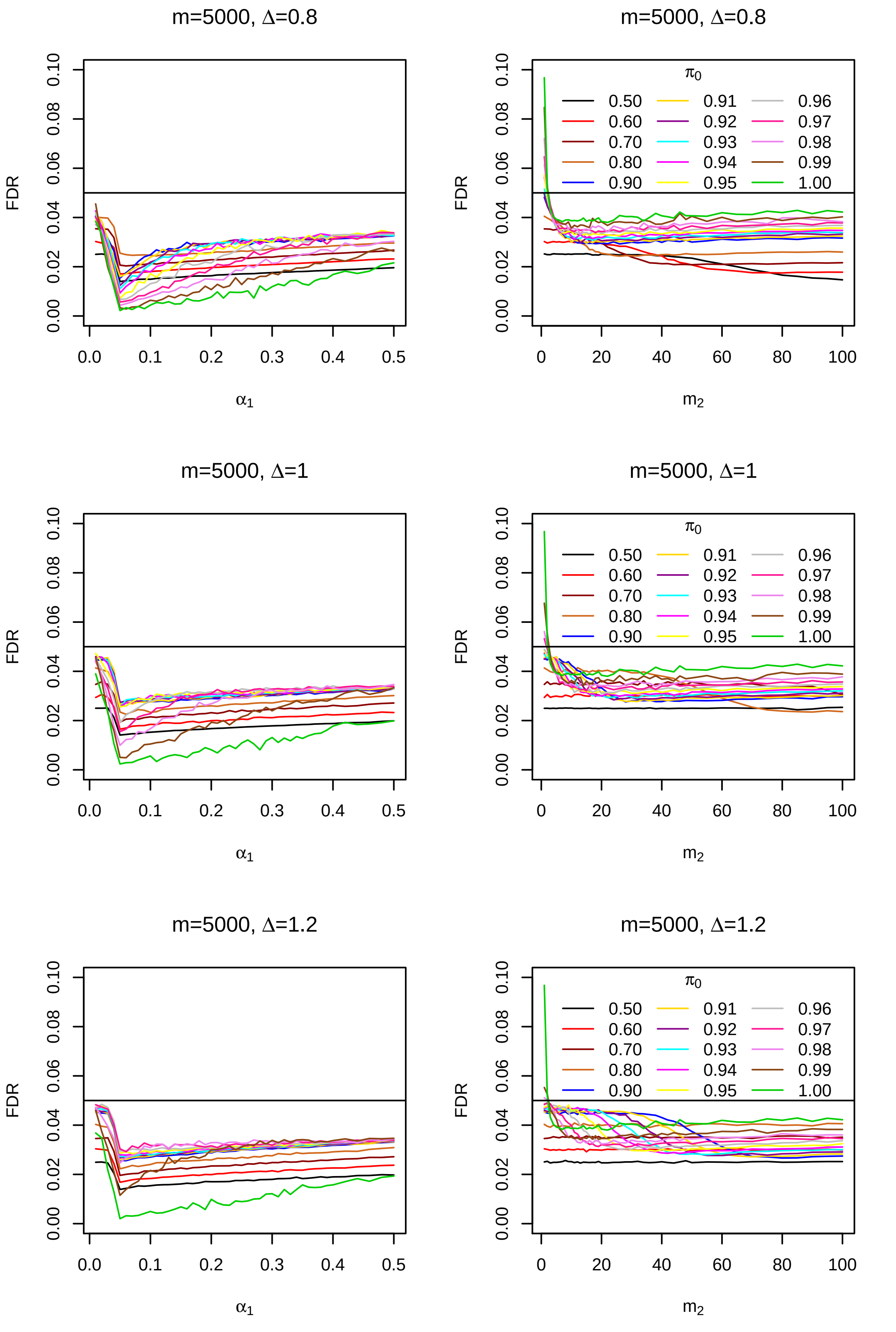
m=5000, Δ=0.8
FDR
α_1
m=5000, Δ=0.8
π_0
0.50 0.60 0.70 0.80 0.90 0.91 0.92 0.93 0.94 0.95 0.96 0.97 0.98 0.99 1.00
FDR
m_2
m=5000, Δ=1
FDR
α_1
m=5000, Δ=1
FDR
m_2
m=5000, Δ=1.2
FDR
α_1
m=5000, Δ=1.2
FDR
m_2

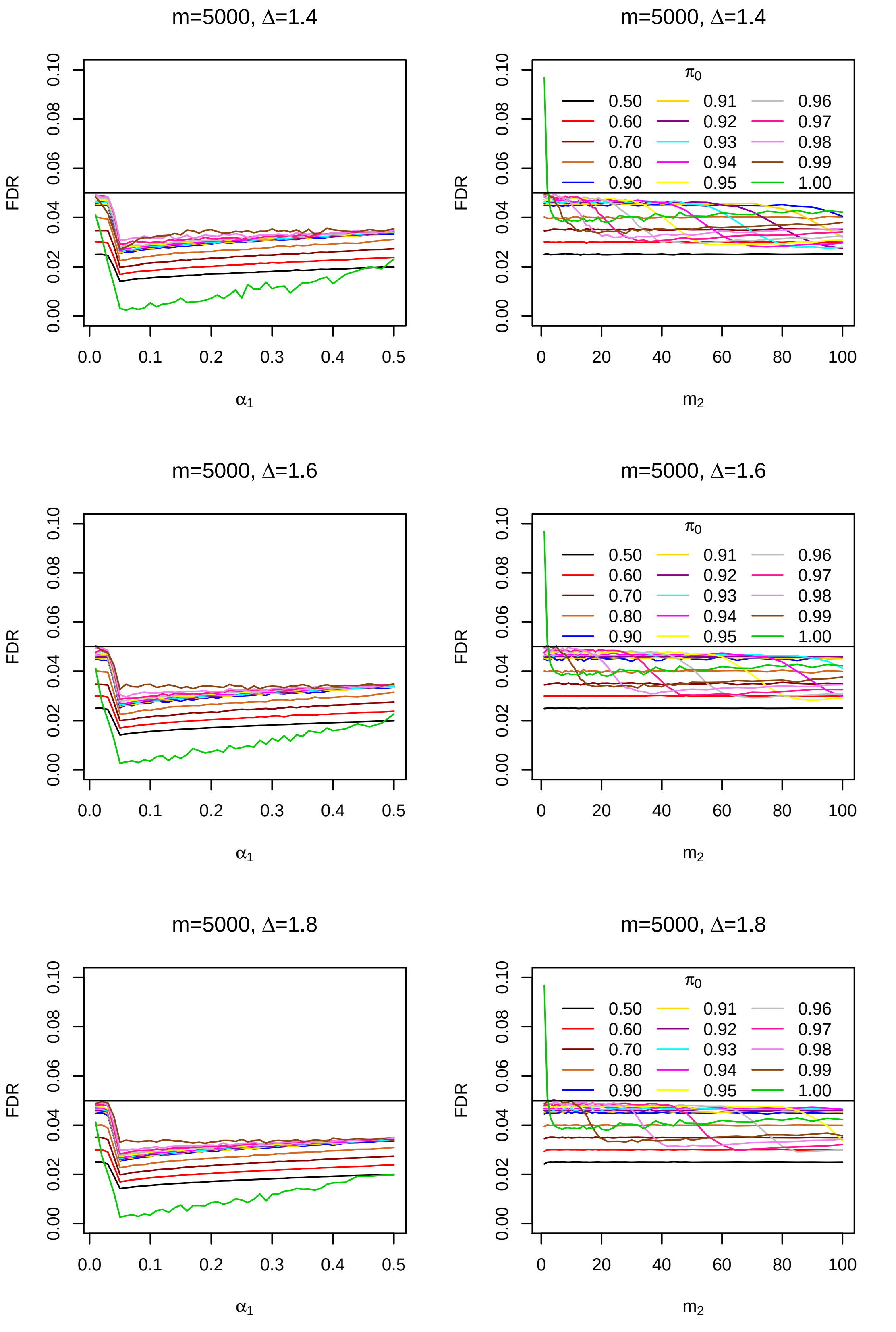

m=5000, Δ=1.4
m=5000, Δ=1.4
m=5000, Δ=1.6
m=5000, Δ=1.6
m=5000, Δ=1.8
m=5000, Δ=1.8
FDR
0.00
0.02
0.04
0.06
0.08
0.10
α1
0.0
0.1
0.2
0.3
0.4
0.5
m2
0
20
40
60
80
100
π0
0.50
0.60
0.70
0.80
0.90
0.91
0.92
0.93
0.94
0.95
0.96
0.97
0.98
0.99
1.00

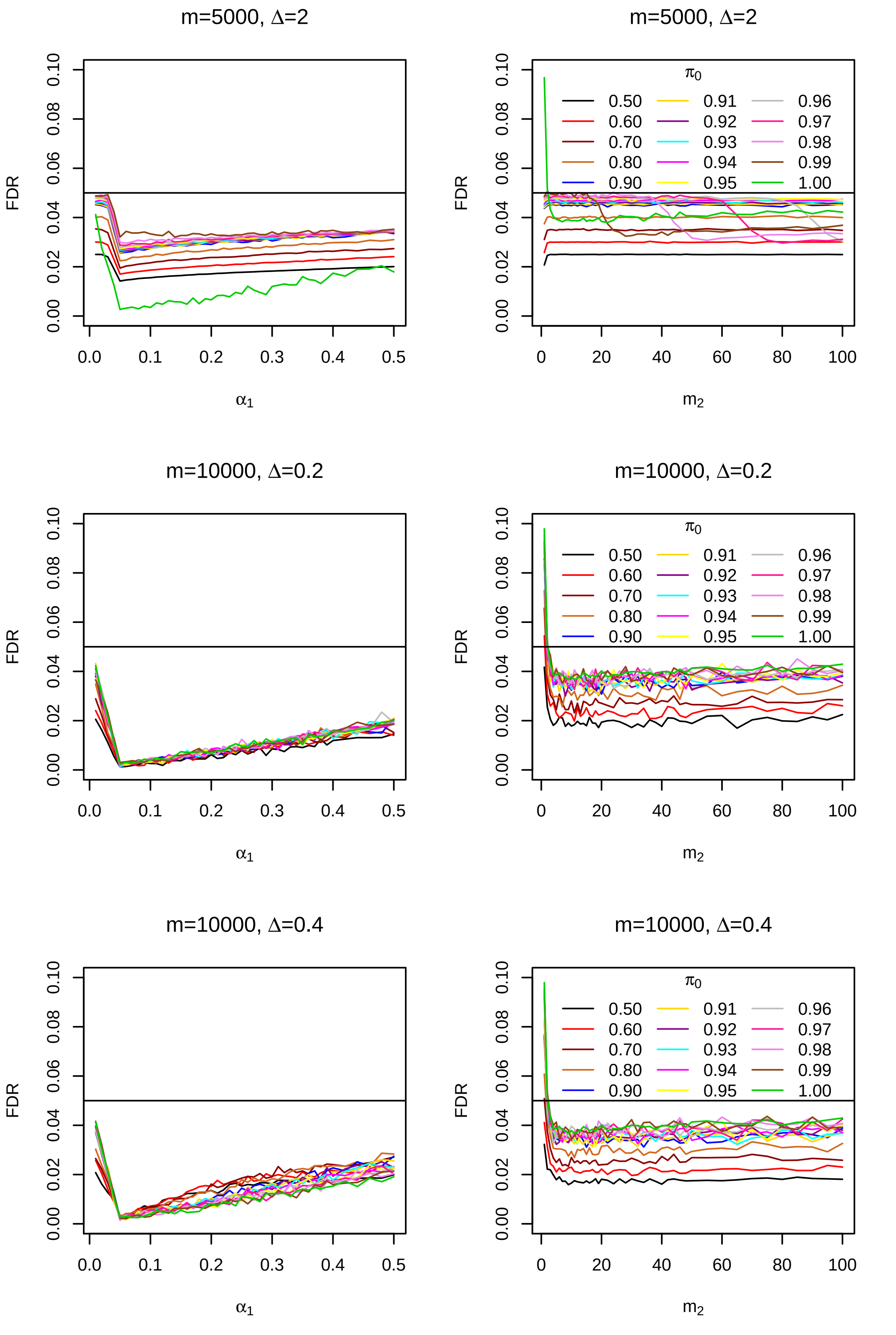
m=5000, Δ=2
m=5000, Δ=2
m=10000, Δ=0.2
m=10000, Δ=0.2
m=10000, Δ=0.4
m=10000, Δ=0.4
FDR
0.00
0.02
0.04
0.06
0.08
0.10
α1
0.0
0.1
0.2
0.3
0.4
0.5
m2
0
20
40
60
80
100
π0
0.50
0.60
0.70
0.80
0.90
0.91
0.92
0.93
0.94
0.95
0.96
0.97
0.98
0.99
1.00

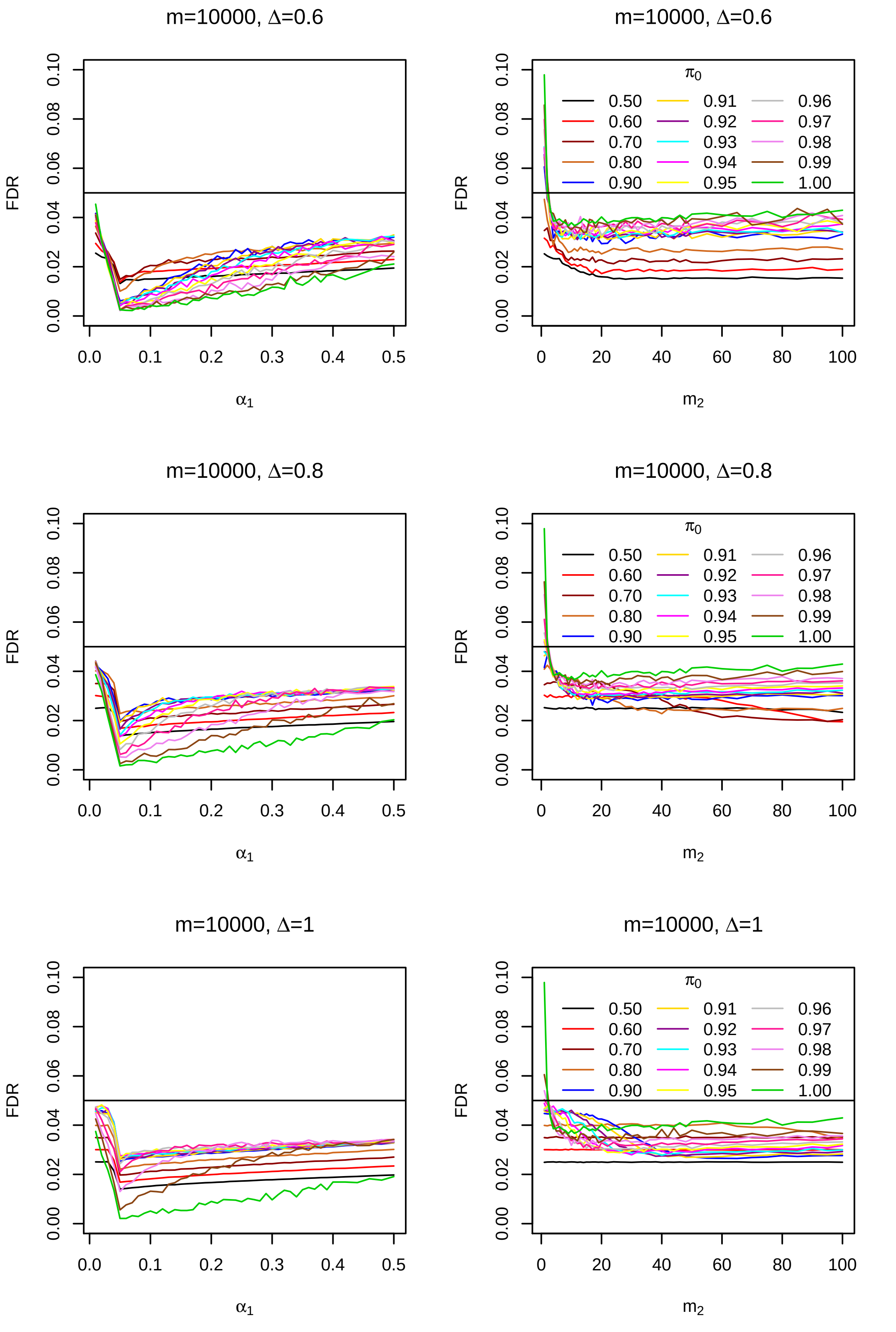
m=10000, Δ=0.6
m=10000, Δ=0.6
m=10000, Δ=0.8
m=10000, Δ=0.8
m=10000, Δ=1
m=10000, Δ=1
FDR
α1
m2
0.00
0.02
0.04
0.06
0.08
0.10
0.0
0.1
0.2
0.3
0.4
0.5
0
20
40
60
80
100
π0
0.50
0.60
0.70
0.80
0.90
0.91
0.92
0.93
0.94
0.95
0.96
0.97
0.98
0.99
1.00

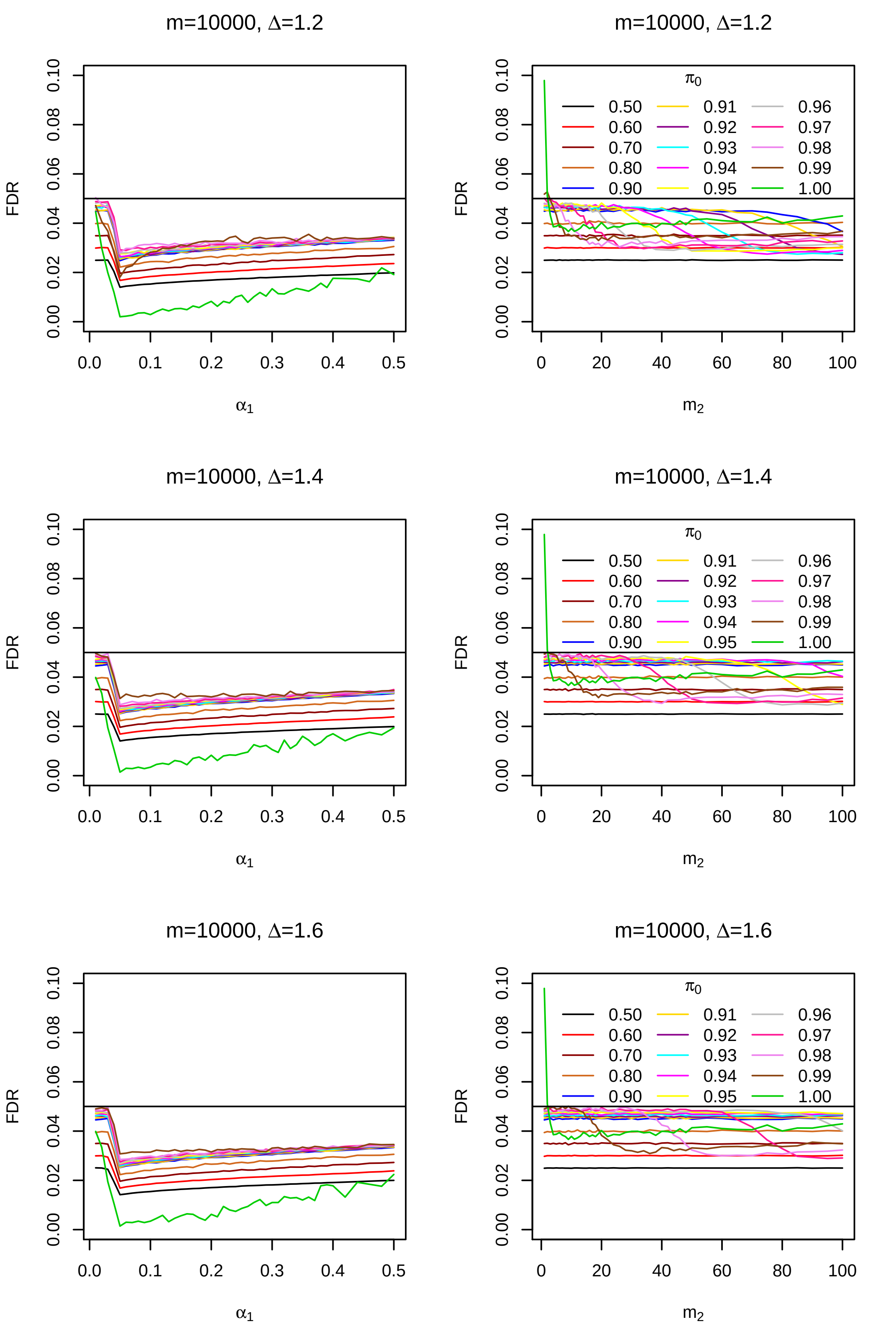
m=10000, Δ=1.2
m=10000, Δ=1.2
m=10000, Δ=1.4
m=10000, Δ=1.4
m=10000, Δ=1.6
m=10000, Δ=1.6
FDR
α_1
m_2
π_0
0.50 0.60 0.70 0.80 0.90
0.91 0.92 0.93 0.94 0.95
0.96 0.97 0.98 0.99 1.00
0.00 0.02 0.04 0.06 0.08 0.10
0.0 0.1 0.2 0.3 0.4 0.5
0 20 40 60 80 100

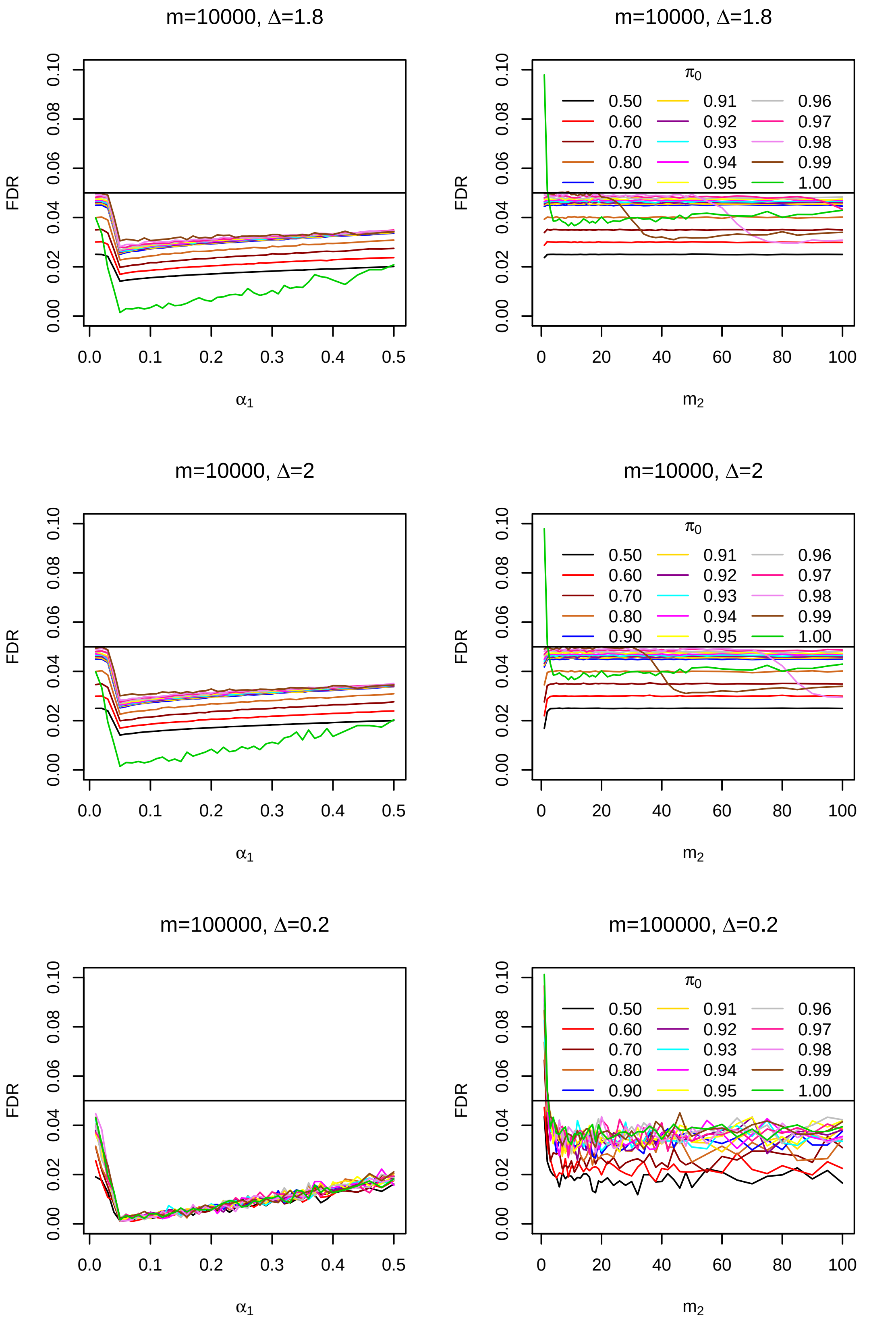

m=10000, Δ=1.8
m=10000, Δ=1.8
m=10000, Δ=2
m=10000, Δ=2
m=100000, Δ=0.2
m=100000, Δ=0.2
FDR
α_1
m_2
0.00 0.02 0.04 0.06 0.08 0.10
0.0 0.1 0.2 0.3 0.4 0.5
0 20 40 60 80 100
π_0
0.50 0.60 0.70 0.80 0.90
0.91 0.92 0.93 0.94 0.95
0.96 0.97 0.98 0.99 1.00

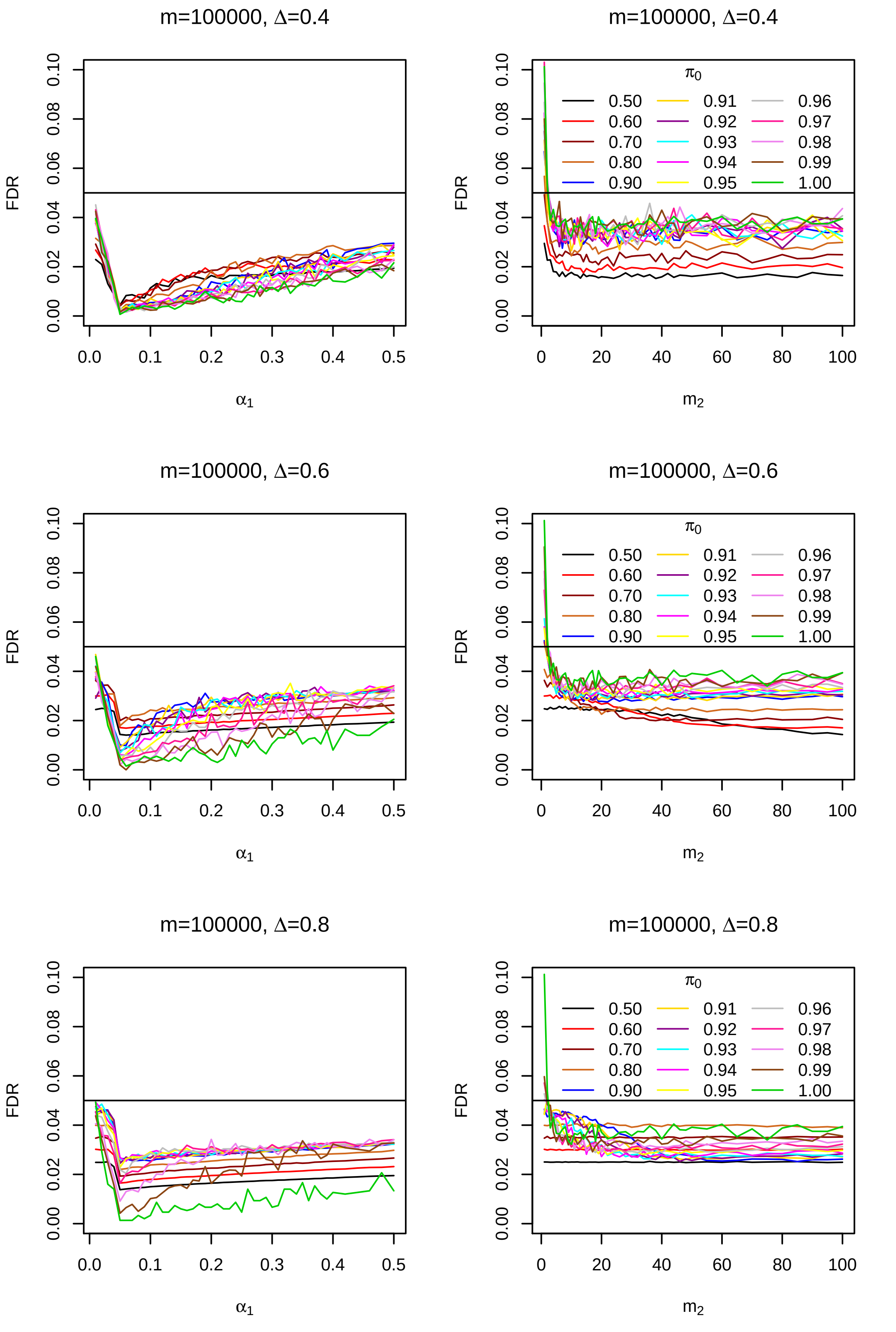
m=100000, Δ=0.4
m=100000, Δ=0.4
m=100000, Δ=0.6
m=100000, Δ=0.6
m=100000, Δ=0.8
m=100000, Δ=0.8
FDR
α1
m2
π0
0.50
0.60
0.70
0.80
0.90
0.91
0.92
0.93
0.94
0.95
0.96
0.97
0.98
0.99
1.00
0.00
0.02
0.04
0.06
0.08
0.10
0.0
0.1
0.2
0.3
0.4
0.5
0
20
40
60
80
100

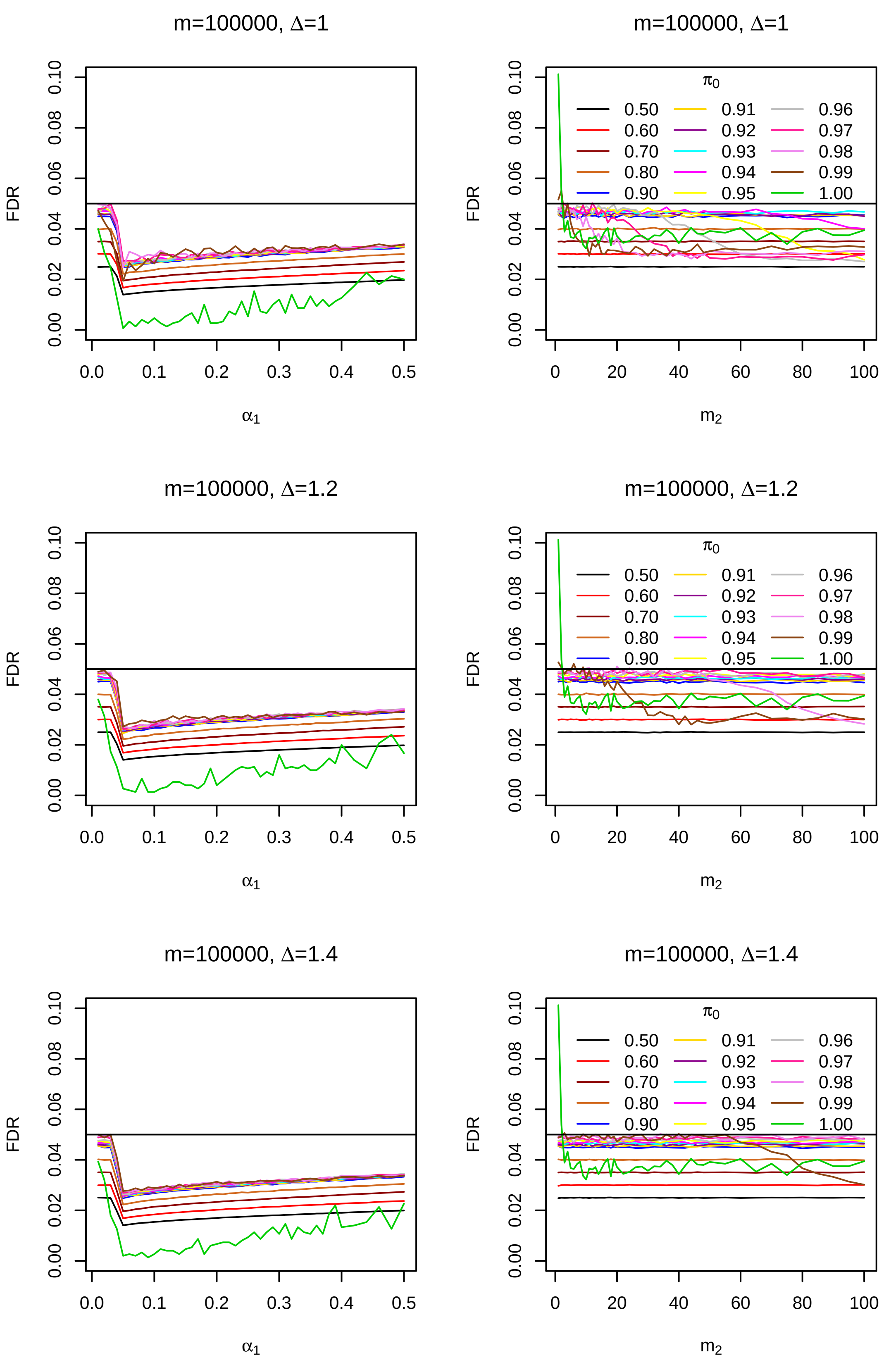

m=100000, Δ=1
m=100000, Δ=1
m=100000, Δ=1.2
m=100000, Δ=1.2
m=100000, Δ=1.4
m=100000, Δ=1.4
FDR
0.00
0.02
0.04
0.06
0.08
0.10
0.0
0.1
0.2
0.3
0.4
0.5
α_1
0
20
40
60
80
100
m_2
π_0
0.50
0.60
0.70
0.80
0.90
0.91
0.92
0.93
0.94
0.95
0.96
0.97
0.98
0.99
1.00

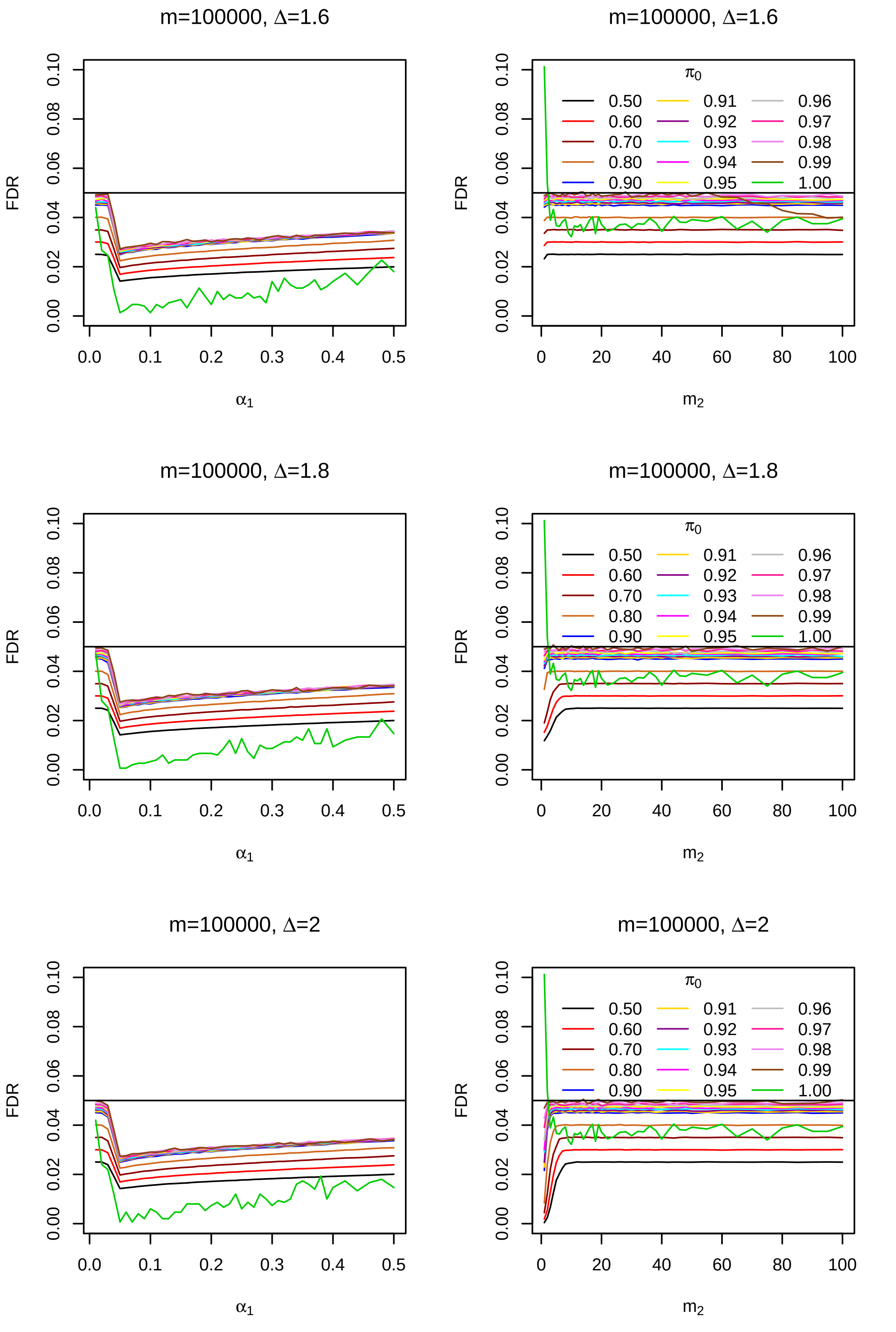
m=100000, Δ=1.6
m=100000, Δ=1.6
m=100000, Δ=1.8
m=100000, Δ=1.8
m=100000, Δ=2
m=100000, Δ=2
FDR
0.00
0.02
0.04
0.06
0.08
0.10
α_1
0.0
0.1
0.2
0.3
0.4
0.5
m_2
0
20
40
60
80
100
π_0
0.50
0.60
0.70
0.80
0.90
0.91
0.92
0.93
0.94
0.95
0.96
0.97
0.98
0.99
1.00
