# Supplementary material for: False discovery rate control in two-stage designs
Source: BMC Bioinformatics. 2012 May 6;13:81. doi: 10.1186/1471-2105-13-81 (PMC3496575; doi:10.1186/1471-2105-13-81)
Supplement: Additional file 2 — Results of a simulation study for two-stage designs where an adaptive test procedures is applied based on an estimator for the proportion of true null hypotheses. [file 1471-2105-13-81-S2.pdf]

## Procedures with adaptive FDR controlling tests

In the preceding simulations the BH method was applied to the sequential p-values to control the FDR. This method is conservative if $\pi_0 < 1$ as it controls the FDR actually at level $\pi_0 \alpha$. Following the suggestion of one of the referees, we additionally considered procedures where the critical value for the final test was chosen such that the estimated FDR is below its nominal level. The estimate of the FDR relies on an estimator of $\pi_0$, $\hat{\pi}_0$ and we considered three estimators in the simulation study: (i) Storey's estimator $\hat{\pi}_0 = \sharp\{p_i > \lambda\}/[(1-\lambda)m]$ where $\lambda$ is a constant $0 < \lambda < 1$ and $\sharp\{p_i > \lambda\}$ denotes the number of p-values exceeding $\lambda$ [1] (see Bibliography below). In the simulations we set $\lambda = 0.5$. (ii) Strimmer's Pfndr estimator [2] which is again a function of the observed p-values. Here a truncated maximum likelihood approach is applied to estimate $\pi_0$. (iii) the oracle estimator for $\pi_0$ which means that we incorporate the true value of $\pi_0$ in the FDR estimation. Again the same scenarios as for the BH test were considered with $m_s = 6$ for the FDRS procedure.

**Integrated design** The adaptive tests are less conservative than the BH test but did not exceed the nominal level (data not shown). The highest FDR values were observed for the Oracle estimator, although for $\pi_0$ close to 1 the differences to the BH procedure were small. For $\pi_0 = 0.5$ the improvement is larger: For example for the FNS design (and $m = 1000$, $\Delta = 1$, $n_1 = 6$, $n_2 = 12$, $m_2 = 50$) the actual FDR increases from 0.022 for the BH procedure to 0.05 (Oracle) or 0.035 (Storey, Pfndr), respectively. There is also an improvement in power in these scenarios: the mean numbers of rejected alternatives increases from 63.15 (BH) to 81 (Storey), 81.35 (Pfndr), and 103.5 (Oracle). Similarly, for the FDRS design (with $\alpha_1 = 0.2$, $m_s = 6$) the actual FDR is increased from 0.017 (BH) to 0.036 (Oracle), 0.024 (Storey) and 0.025 (Pfndr), respectively. Thus, the FDRS procedure is still conservative in this setting because of the modification (setting $m_s = 6$) and because the $\pi_0$ estimators are negatively biased. The mean numbers of rejected alternatives increases from 150 (BH) to 183.5 (Storey), 184.5 (Pfndr), and 222.75 (Oracle). Thus, in the scenario with a low $\pi_0$ of 0.5 for both designs the adaptive FDR estimation can increase the power considerably while maintaining the actual FDR level. Tables 1 and 2 show the mean number of rejected alternatives for scenarios with $\pi_0$ close to 1 as described in the manuscript.

**Pilot design** Note that the FDRS pilot design is already an adaptive procedure. Because the selection of hypotheses in the first stage is based on a test controlling the FDR at level $\alpha_1$, $\alpha_1$ is a (conservative) estimate for the proportion of true null hypotheses among the selected hypotheses. In the FDRS method the nominal level $\alpha/\alpha_1$ is applied at the second stage such that the BH procedure controls the FDR at level $\alpha$.

For the pilot design we performed additional simulations applying the adaptive tests based on the modified Storey estimator (as described in [3]) and the Oracle estimator. With the FDRS rule sometimes only very few hypotheses are selected for the second stage. The same is true given a small $m_2$ for the FNS procedure. Therefore, we used the modified Storey estimator (that is conservative for low number of hypotheses) and did not include the Pfndr estimator in the simulation study, because it fails to give an estimate in settings with too few hypotheses. As in the integrated design the adaptive pilot design tests are less conservative than the BH tests but did not exceed the nominal level (data not shown) and for $\pi_0$ close to 1 the differences to the BH procedure were small (see Tables 3 and 4 below for the mean number of rejected alternatives).

**Table 1 - FNS Design: Integrated Design**

The mean number of rejected alternatives for the integrated design for the Oracle (Or), Storey (St) and Pfndr (Pf) methods for independent test statistics and $\alpha = 0.05$, $n_1 = 6$, $n_2 = 12$.

**Table 2 - FDRS Design: Integrated Design**

The mean number of rejected alternatives for the integrated design for the Oracle (Or), Storey (St) and Pfndr (Pf) methods for independent test statistics and $\alpha = 0.05$, $n_1 = 6$, $n_2 = 12$.

**Table 3 - FNS Design: Pilot Design**

The mean number of rejected alternatives for the pilot design for the Oracle (Or) and Storey (St) methods for independent test statistics and $\alpha = 0.05$, $n_1 = 6$, $n_2 = 12$.

**Table 4 - FDRS Design: Pilot Design**

The mean number of rejected alternatives for the pilot design for the Oracle (Or) and Storey (St) methods for independent test statistics and $\alpha = 0.05$, $n_1 = 6$, $n_2 = 12$.

| | | $m = 1000$ | | | | | | $m = 10000$ | | | | | | $m = 100000$ | | | | | |
|---|---|---|---|---|---|---|---|---|---|---|---|---|---|---|---|---|---|---|---|
| $m_2$ | $\pi_0$ | $\Delta = 1$ | | | $\Delta = 1.6$ | | | $\Delta = 1$ | | | $\Delta = 1.6$ | | | $\Delta = 1$ | | | $\Delta = 1.6$ | | |
| | | Or | St | Pf | Or | St | Pf | Or | St | Pf | Or | St | Pf | Or | St | Pf | Or | St | Pf |
| $0.01m$ | .95 | 6.1 | 6.1 | 6.1 | 15.5 | 15.5 | 15.5 | 58.9 | 58.8 | 58.8 | 144.0 | 143.6 | 143.7 | 586.1 | 585.1 | 585.1 | 1427.5 | 1423.5 | 1423.1 |
| $0.01m$ | .99 | 1.8 | 1.8 | 1.8 | 5.0 | 5.0 | 5.0 | 13.3 | 13.3 | 13.3 | 41.9 | 41.9 | 41.9 | 126.9 | 126.9 | 126.9 | 407.5 | 407.5 | 407.5 |
| $0.05m$ | .95 | 12.6 | 12.6 | 12.6 | 26.7 | 26.7 | 26.7 | 118.5 | 118.1 | 118.1 | 260.5 | 260.4 | 260.4 | 1175.1 | 1171.3 | 1171.7 | 2594.0 | 2593.1 | 2593.2 |
| $0.05m$ | .99 | 2.8 | 2.8 | 2.8 | 6.2 | 6.2 | 6.2 | 19.8 | 19.8 | 19.8 | 53.8 | 53.8 | 53.8 | 188.8 | 188.6 | 188.6 | 524.0 | 523.9 | 523.9 |
| $0.1m$ | .95 | 15.0 | 15.0 | 15.0 | 30.2 | 30.2 | 30.1 | 141.1 | 140.6 | 140.4 | 294.5 | 294.4 | 294.4 | 1400.2 | 1395.1 | 1391.5 | 2933.0 | 2931.6 | 2930.5 |
| $0.1m$ | .99 | 3.1 | 3.1 | 3.1 | 6.5 | 6.5 | 6.5 | 22.1 | 22.1 | 22.0 | 57.1 | 57.1 | 57.1 | 210.4 | 210.2 | 210.0 | 554.9 | 554.9 | 554.8 |

| $\alpha_1$ | $\pi_0$ | $m=1000$ | | | | | | $m=10000$ | | | | | | $m=100000$ | | | | | |
|---|---|---|---|---|---|---|---|---|---|---|---|---|---|---|---|---|---|---|---|
| | | $\Delta=1$ | | | $\Delta=1.6$ | | | $\Delta=1$ | | | $\Delta=1.6$ | | | $\Delta=1$ | | | $\Delta=1.6$ | | |
| | | Or | St | Pf | Or | St | Pf | Or | St | Pf | Or | St | Pf | Or | St | Pf | Or | St | Pf |
| 0.1 | .95 | 2.6 | 2.6 | 2.6 | 18.5 | 18.5 | 18.5 | 18.6 | 18.2 | 18.2 | 174.2 | 173.6 | 173.7 | 175.3 | 171.4 | 171.5 | 1727.1 | 1721.7 | 1721.8 |
| | .99 | 0.4 | 0.4 | 0.4 | 3.3 | 3.3 | 3.3 | 1.3 | 1.3 | 1.3 | 23.2 | 23.2 | 23.2 | 7.5 | 7.5 | 7.5 | 220.5 | 220.3 | 220.3 |
| 0.2 | .95 | 4.5 | 4.4 | 4.4 | 22.0 | 22.9 | 22.9 | 35.8 | 35.1 | 35.1 | 209.2 | 208.6 | 208.6 | 346.1 | 338.6 | 338.6 | 2075.7.1 | 2069.1 | 2069.2 |
| | .99 | 0.6 | 0.6 | 0.6 | 3.9 | 3.9 | 3.9 | 2.3 | 2.3 | 2.3 | 28.9 | 28.9 | 28.9 | 16.0 | 15.9 | 15.9 | 276.6 | 276.4 | 276.5 |
| 0.5 | .95 | 9.8 | 9.6 | 9.6 | 27.8 | 27.7 | 27.8 | 86.0 | 84.0 | 84.0 | 268.6 | 267.6 | 267.6 | 847.6 | 826.8 | 827.2 | 2670.2 | 2660.2 | 2660.4 |
| | .99 | 1.3 | 1.3 | 1.3 | 5.1 | 5.1 | 5.1 | 6.2 | 6.2 | 6.2 | 40.0 | 40.0 | 40.0 | 52.0 | 51.4 | 51.5 | 384.7 | 383.0 | 383.1 |

| $m_2$ | $\pi_0$ | $m = 1000$ | | | | $m = 10000$ | | | | $m = 100000$ | | | |
|---|---|---|---|---|---|---|---|---|---|---|---|---|---|
| | | $\Delta = 1$ | | $\Delta = 1.6$ | | $\Delta = 1$ | | $\Delta = 1.6$ | | $\Delta = 1$ | | $\Delta = 1.6$ | |
| | | Or | St | Or | St | Or | St | Or | St | Or | St | Or | St |
| $0.01m$ | .95 | 6.0 | 5.7 | 9.9 | 9.8 | 56.8 | 55.9 | 98.6 | 98.5 | 564.2 | 557.6 | 985.4 | 985.4 |
| $0.01m$ | .99 | 1.7 | 1.7 | 4.9 | 4.9 | 11.6 | 11.5 | 41.4 | 41.4 | 110.8 | 110.4 | 401.9 | 401.8 |
| $0.05m$ | .95 | 11.2 | 11.0 | 26.3 | 26.2 | 103.9 | 102.8 | 255.8 | 255.4 | 1028.5 | 1018.7 | 2545.3 | 2542.2 |
| $0.05m$ | .99 | 2.3 | 2.3 | 6.0 | 6.0 | 15.0 | 14.9 | 51.2 | 51.2 | 141.8 | 141.4 | 496.2 | 496.0 |
| $0.1m$ | .95 | 12.5 | 12.3 | 29.0 | 28.9 | 115.4 | 114.3 | 282.5 | 282.2 | 1142.2 | 1132.3 | 2810.1 | 2806.8 |
| $0.1m$ | .99 | 4.9 | 4.9 | 6.1 | 6.1 | 15.2 | 15.1 | 52.7 | 52.7 | 143.1 | 142.8 | 510.3 | 510.2 |

| | | $m = 1000$ | | | | $m = 10000$ | | | | $m = 100000$ | | | |
|---|---|---|---|---|---|---|---|---|---|---|---|---|---|
| $\alpha_1$ | $\pi_0$ | $\Delta = 1$ | | $\Delta = 1.6$ | | $\Delta = 1$ | | $\Delta = 1.6$ | | $\Delta = 1$ | | $\Delta = 1.6$ | |
| | | Or | St | Or | St | Or | St | Or | St | Or | St | Or | St |
| 0.1 | .95 | 2.7 | 2.7 | 18.6 | 18.5 | 18.9 | 18.5 | 174.8 | 174.2 | 177.9 | 173.9 | 1733.5 | 1728.1 |
| | .99 | 0.4 | 0.4 | 3.3 | 3.3 | 1.3 | 1.3 | 23.2 | 23.2 | 7.4 | 7.3 | 220.9 | 220.7 |
| 0.2 | .95 | 4.7 | 4.5 | 22.3 | 22.1 | 37.4 | 36.3 | 212.1 | 211.3 | 363.3 | 354.6 | 2105.3 | 2098.3 |
| | .99 | 0.7 | 0.7 | 3.9 | 3.9 | 2.5 | 2.4 | 29.2 | 29.2 | 16.4 | 16.2 | 278.7 | 278.5 |
| 0.5 | .95 | 9.9 | 9.5 | 27.9 | 27.8 | 87.9 | 85.2 | 269.8 | 268.6 | 866.8 | 840.5 | 2683.7 | 2672.0 |
| | .99 | 1.4 | 1.3 | 5.1 | 5.0 | 6.4 | 6.2 | 40.2 | 40.1 | 53.4 | 52.8 | 385.5 | 385.0 |

# Bibliography

[1] J. D. Storey. A direct approach to false discovery rates. *J R Statist Soc B*, 64:479–498, 2002.

[2] K. Strimmer. A unified approach to false discovery rate estimation. *BMC Bioinformatics*, 9:303317, 2008.

[3] S. Zehetmayer, P. Bauer, and M. Posch. Two-stage designs for experiments with a large number of hypotheses. *Bioinformatics*, 21:3771–3777, 2005.
