# Supplementary material for: False discovery rate control in two-stage designs
Source: BMC Bioinformatics. 2012 May 6;13:81. doi: 10.1186/1471-2105-13-81 (PMC3496575; doi:10.1186/1471-2105-13-81)
Supplement: Additional file 3 — Two single-stage designs are compared to the results: For the first single-stage design the sample size for each hypothesis is n1, for the second design the sample size is n1 + n2. For the first design we compare the gain in power of the integrated design and for the second design the attention lies on the reduction in costs. [file 1471-2105-13-81-S3.pdf]

Comparison to single-stage designs

It is well known that two-stage procedures are typically more powerful than single-stage designs with the same total number of observations [1-8].In this section we show how this finding translates to the newly proposed tests for the considered two-stage designs. We compare the two-stage designs with two different single-stage designs: For the first single-stage design the sample size for each hypothesis is n_1 ("n_1"-Design), the sample size of the first stage of the two-stage design. Here it is examined if a two-stage design outperforms the single-stage design and under which circumstances the power can be considerably increased. In the second single-stage design the total sample size for each hypothesis is $n_1 + n_2$ ("$n_1 + n_2$"-Design). Here, a gain in power (or at least equal power) is expected and the main attention lies on the reduction in costs in terms of the total number of single observations (e.g., costs = $(n_1 + n_2)m$ for the single-stage design and costs = $n_1 m + n_2 m_2$ for the FNS design).

The table below shows the mean number of rejected alternatives for the two single-stage designs for the investigated main scenarios (independent data). "n_1"-Design: The FNS design has a higher power than the single-stage designs for all scenarios but for small m_2 and large $\Delta = 1.6$ where the FNS design rejects the same mean number of alternatives (and the total number of single observations for both designs are very similar). The FDRS design is always more powerful than the single-stage design. "$n_1 + n_2$"-Design: The single-stage design rejects more alternatives than both two-stage designs in all considered scenarios. However, in the considered scenarios the single-stage designs requires between 2.5 and 2.94 (2.7 and 3) times more observations than the FNS (FDRS) design.

Table: Single-stage design

The mean number of rejected alternatives for the two considered single-stage designs with $n = n_1 + n_2$ and $n = n_1$ (in parentheses), respectively, for independent scenarios with $\alpha = 0.05$, $n_1 = 6$, $n_2 = 12$ (20000 simulation runs per scenario for $m = 1000$, $m = 10000$; 10000 simulation runs for $m = 100000$).

	$m = 1000$		$m = 10000$		$m = 100000$	
	$\Delta = 1$	$\Delta = 1.6$	$\Delta = 1$	$\Delta = 1.6$	$\Delta = 1$	$\Delta = 1.6$
$\pi_0 = 0.95$	18.7 (1.56)	34 (15.4)	176.5 (9)	333 (141.9)	1749 (81.5)	3320 (1406.8)
$\pi_0 = 0.99$	3.5 (0.3)	6.8 (2.7)	25 (1)	69.8 (18.4)	240 (3.6)	593.9 (173.7)
